# MicroFace maps microglial morphology remodeling, revealing spatial zones and bifurcated trajectories during brain microinjury recovery

**DOI:** 10.1016/j.isci.2026.116485

**Published:** 2026-06-23

**Authors:** Vatsal D. Jariwala, Shreya Ponnamma, Vidhya M. Ravi, Jürgen Beck, Ulrich G. Hofmann, Kevin Joseph

**Affiliations:** 1 Laboratory for NeuroEngineering, Department of Neurosurgery, Medical Center-University of Freiburg, Freiburg, Germany; 2 3D Brain Models Lab, Department of Neurosurgery, Medical Center-University of Freiburg, Freiburg, Germany; 3 Department of Neurosurgery, Medical Center-University of Freiburg, Freiburg, Germany; 4 Faculty of Medicine, University of Freiburg, Freiburg, Germany; 5 Freiburg Institute for Advanced Science (FRIAS), University of Freiburg, Freiburg, Germany; 6 Neuroelectronic Systems, Department of Neurosurgery, Medical Center-University of Freiburg, Freiburg, Germany

**Keywords:** health sciences, biological sciences, cellular physiology, cell biology, functional aspects of cell biology

## Abstract

Microglia undergo morphological remodeling in response to brain injury, yet large-scale quantification of these changes remains limited. Here, we present MicroFace, an automated image analysis pipeline for high-throughput reconstruction and morphometric profiling of microglia from immunofluorescence images. We applied MicroFace to 279,510 microglia from the rodent cortex following localized microinjury induced by neural probe implantation. Spatiotemporal analysis revealed distinct morphotypes spanning ramified and amoeboid states, organized along spatial gradients relative to the injury site and evolving over time. We identify a bifurcated response characterized by divergent intermediate morphologies, including reactive transient cells and elongated rod-like microglia enriched near the injury. Integration with transcriptomic datasets suggests that rod-like microglia represent a morphologically and transcriptionally distinct subset associated with immunomodulatory functions. These findings define dynamic and heterogeneous microglial adaptations to brain injury and highlight morphology as a key indicator of functional state.

## Highlights

• MicroFace maps large-scale spatiotemporal continuum of microglial morphologies
• Microglial morphology is spatially organized into 3 zones around brain microinjury
• Microglial density and amoeboid morphology peak at 1 WPI and then normalize
• Rod-like microglia form a distinct transient population near injury

## Graphical abstract

**Figure:**
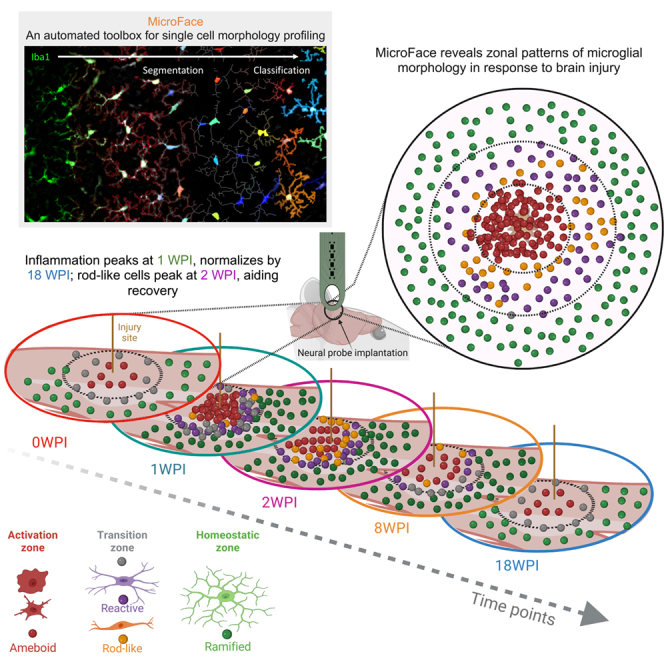

## Introduction

Rapid advancements in neurotechnology have catalyzed the development of innovative invasive approaches for monitoring neuronal activity. Central to this progress are implantable neural interfaces, which have become indispensable tools in neuroscience research and clinical practice. These devices, capable of recording and stimulating electrical activity within the central nervous system, play a crucial role in elucidating neural mechanisms and enhancing therapeutic interventions.^1^ Neural interfaces typically consist of invasive probes that can record or stimulate electrical activity within the central nervous system.^2^ Several probe configurations have been developed, including penetrating electrodes^3^ inserted directly into neural tissue, surface electrodes^4^ positioned on the cortical surface, and multi-electrode arrays that allow simultaneous recording from multiple neuronal populations.^5^

The material properties of neural probes strongly influence their performance and their interactions with surrounding tissue. Silicon probes are widely used due to their mechanical stability and high signal-to-noise ratio,^6^ whereas metallic electrodes made of platinum/titanium are commonly employed in stimulation devices because of their favorable electrical properties.^7^^,^^8^ More recently, flexible polymer-based probes have been developed to better match the mechanical properties of brain tissue and reduce chronic tissue damage caused by mechanical mismatch between rigid implants and the surrounding neural environment.^9^^,^^10^ Despite these advances, implantation of neural probes inevitably disrupts the surrounding brain tissue and triggers a cascade of biological responses. The invasive nature of probe implantation disrupts brain networks, causing highly localized traumatic brain injury (TBI) and oxidative stress and initiating acute inflammatory responses.^11^^,^^12^ TBI involves two phases: acute and chronic. The acute injury phase occurs immediately following mechanical trauma to the brain tissue, such as during probe implantation, resulting in structural damage and initiating a cascade of cellular and molecular events. The chronic injury phase lasts from hours to weeks after the initial trauma, inflammation, and apoptosis.^13^^,^^14^ These processes lead to brain edema, neurodegeneration, and cognitive deficits leading to activation of the innate immune system, particularly by inflammatory cells such as microglia and astrocytes, which release cytokines and chemokines, attracting other immune cells to the injury site.^15^^,^^16^ The glial response results in the formation of a sequestering scar within 1–2 weeks of injury,^17^ serving as a protective barrier but also impeding neural cell movement and nutrient flow, exacerbating neurodegeneration around the injury.^18^ In addition to the effect of the localized TBI, the long-term presence of these probes often results in a persistent foreign body reaction.^19^ Mechanical mismatch between the probe and brain tissue can lead to relative movement, further introducing stress to the tissue, compromising functional efficacy by diminishing electrode performance as we have reported previously.^20^

Microglia play a pivotal role in the regulation of neuronal development, synaptic pruning, and maintenance of local homeostasis and respond to changes in neural activity.^21^ Upon activation due to injury or infection, microglia transition from their resting state to an activated state characterized by changes in morphology, increased phagocytic activity, and cytokine secretion.^22^^,^^23^ Microglia activation in response to environmental disturbances occurs across a spectrum of phenotypes, ranging from pro-inflammatory toanti-inflammatory states.^24^ Pro-inflammatory microglia, associated with classical activation, release inflammatory cytokines and chemokines, contributing to neuroinflammation and neuronal damage. In contrast, anti-inflammatory microglia, associated with alternative activation, promote tissue repair and neuroprotection,^24^^,^^25^ also supported by high-dimensional transcriptomic readouts.^26^^,^^27^

Although extensive transcriptomic characterization has been carried out in microglia post-injury, there lacks a morphological identification of specific injury response phenotypes post-injury. Microglial morphology is a sensitive indicator of cellular activation and functional state. In healthy tissue, microglia display highly ramified morphologies with complex branching processes that support continuous surveillance of the neural environment.^28^^,^^29^ Following injury, these cells have been reported to adopt more compact morphologies characterized by enlarged somas and reduced branching complexity.^30^^,^^31^ Quantitative analysis of these structural changes could provide valuable insight into the spatial organization and functional dynamics of microglial responses.^32^ However, systematic characterization of microglial morphology remains challenging since existing approaches rely largely on manual or semi-automated segmentation methods that are labor intensive, prone to observer bias, and difficult to scale to large imaging datasets.^33^ To overcome these limitations, we developed MicroFace, an automated image analysis pipeline for rapid and minimally biased quantification of microglial morphology from immunofluorescence (IF) images. Applying MicroFace to microglia across spatial distinct regions and time points after flexible neural probe implantation, we identify spatially organized microglial morphotypes surrounding the implant site. These analyses reveal gradients of morphological states extending from the injury interface into surrounding tissue, providing new insight into the structural organization of microglial responses during chronic foreign body reactions to neural implants.

## Results

### Automated workflow for large-scale microglial morphometric analysis

**Figure 1:**
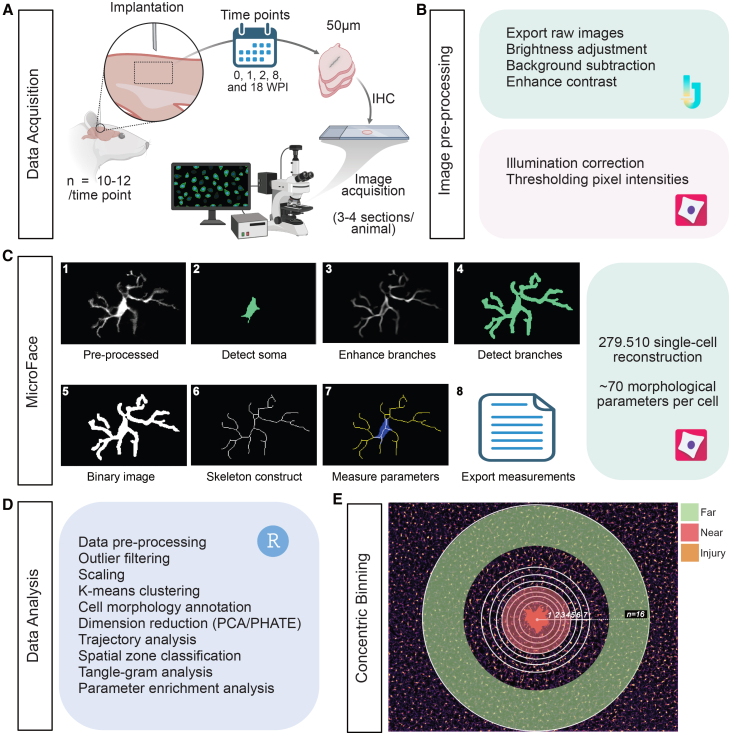
Experimental workflow for imaging, reconstruction, and quantitative analysis of microglial morphology after neural probe implantation (A) Overview of the experimental design. Rats were implanted with flexible neural probes in the motor cortex and maintained for different post-implantation intervals (0, 1, 2, 8, and 18 weeks post-implantation [WPI]). At each time point, animals were euthanized and brains were collected for immunohistochemistry. Coronal brain sections (50 μm thickness) were stained for microglia and imaged to capture cellular morphology surrounding the probe track. (B) Image pre-processing workflow. Raw immunofluorescence images were processed using ImageJ and CellProfiler to perform illumination correction, background adjustment, and preparation of images for segmentation and morphological analysis. (C) MicroFace morphology reconstruction pipeline. Sequential image-analysis steps used to identify individual microglia and reconstruct their morphology from immunofluorescent images. The pipeline includes cell detection, soma identification, process extraction, skeletonization, and feature extraction. Across all images, ∼279,510 microglia cells were reconstructed, and ∼70 morphological features were quantified per cell. (D) Data processing and statistical analysis. Extracted morphological features were compiled and analyzed using R Studio for downstream statistical analysis, clustering, and morphotype classification. (E) Spatial analysis around the injury site. Each image was divided into concentric radial bins (radius = 140 pixels) centered on the probe injury site to quantify spatial patterns in microglial morphology. 16 bins were generated per image. The injury center is indicated in orange, bins closest to the injury are shown in red, and bins farther from the injury site are shown in green.

The complex morphology of microglia is essential for their roles in phagocytosis, migration, and cytokine release.^34^ Quantifying these morphological features at large scale remains challenging because manual tracing and annotation of microglial structures is time-consuming and difficult to apply across large datasets. To address this limitation, we developed MicroFace, an automated image segmentation and analysis workflow designed to systematically reconstruct and quantify microglial morphology from IF images (Figures 1A–1D). In this study, IBA1-stained IF images were acquired from the motor cortex of female rats following implantation of flexible neural probes and analyzed across multiple time points (0, 1, 2, 8, and 18 weeks post-implantation [WPI]; Figure 1A). The main processing steps of the MicroFace pipeline, including image preprocessing, illumination correction, cell segmentation, skeleton reconstruction, and feature extraction, are outlined in Figures 1 and S1 and described in detail in the STAR Methods section.

### Validation of the MicroFace segmentation and morphometric quantification pipeline

**Figure 2:**
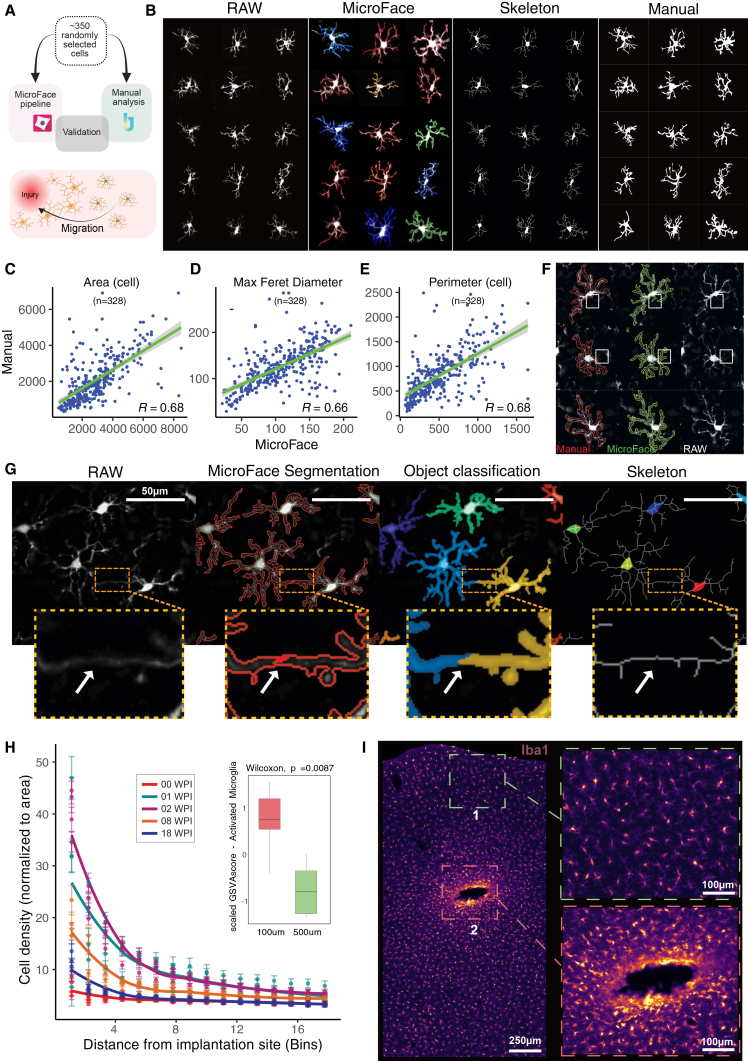
Validation of the MicroFace segmentation pipeline and the spatial differences in microglial density with respect to the injury (A) Schematic of the validation strategy. (B) Representative examples (*n* = 15) of MicroFace segmentation. Rows show raw IBA1 immunofluorescence images of microglia, the corresponding MicroFace segmentation masks, skeletonized reconstructions of cellular processes, and examples of manually segmented cells used for comparison. (C–E) Correlation between MicroFace and manual segmentation measurements. Scatterplots compare morphological features extracted from MicroFace (*x* axis) with values obtained from manual segmentation (*y* axis). (C) Cell area (*n* = 326 cells, r = 0.68). (D) Maximum Feret diameter (*n* = 326 cells, r = 0.66). (E) Cell perimeter (*n* = 326 cells, r = 0.68). Each point represents a single microglial cell. (F) Examples (*n* = 3) highlighting differences between segmentation approaches. Representative raw images are shown alongside MicroFace and manual segmentation outputs. In some cases, fine distal branches detected by MicroFace are not captured during manual tracing. (G) Segmentation and object separation workflow of MicroFace. Insets illustrate examples of adjacent microglial processes and how intensity-based separation are used to distinguish processes belonging to neighboring cells. (H) Spatial distribution of microglial density around the implant site (*n* = 279,510 cells). Inset: GSVA analysis of activated microglia signatures across spatially distinct regions. (I) Representative IBA1 immunofluorescence images illustrating spatial differences in microglial distribution. The top inset shows microglia located distal to the implant site with more evenly distributed morphology, whereas the bottom inset shows the region adjacent to the injury with increased microglial density.

To evaluate the accuracy of the MicroFace segmentation pipeline, we compared automated measurements with manual segmentation performed using Fiji.^35^ For this validation, approximately 350 microglia were randomly selected from the dataset and independently segmented using both approaches (Figures 2A and 2B). From these cells, morphological parameters that could be reliably extracted across both methods were quantified and compared. Correlation analysis showed positive relationships across the tested parameters, including cell area (R = 0.68), maximum Feret diameter (R = 0.66), cell perimeter (R = 0.68), cell radius (R = 0.58), convex area (R = 0.67), and solidity (R = 0.72) (Figures 2C–2E; additional parameters in Figure S2A). Visual inspection of segmentation outputs further showed that MicroFace was able to detect fine distal branches that were sometimes missed during manual tracing (Figures 2F and S2B). This potentially reflects the difficulty of consistently identifying thin processes during manual annotation, particularly when large numbers of cells are analyzed. Because microglial processes frequently overlap in regions close to the injury site, we also evaluated the ability of MicroFace to separate neighboring cells (Figure 2G). The segmentation algorithm uses intensity gradients and smooth filtering to distinguish adjacent branches belonging to different cells. This approach allowed reliable separation of processes from neighboring microglia, even when cells were in close proximity (Figures 2G, S3A, and S3B). In addition, the pipeline was able to resolve closely positioned somas in densely populated regions near the injury site (Figures S3C and S3D), demonstrating that MicroFace provides a scalable approach for quantifying microglial morphology in large image datasets.

To characterize the cellular response to probe implantation, we quantified the spatial distribution of Iba1-positive cells around the injury site. The spatial positioning of the cell was assigned to concentric radial bins extending outward from the implant track (Figure 1E). Consistent with previous reports^36^ of microglial responses to brain injury, we observed increased microglial density in regions adjacent to the implant at intermediate time points (Figure 2H). Specifically, Iba1-positive cell density near the injury site increased by approximately 60% at 1 WPI compared with 0 WPI. Elevated cell numbers persisted at 2 and 8 WPI, with approximately 50% higher density relative to the acute condition. In contrast, regions located farther from the injury site showed relatively stable microglial density across time points. By 18 WPI, the spatial distribution of microglia had largely returned to levels comparable to 0 WPI, suggesting partial restoration of tissue homeostasis regardless of the electrode dimensions (Figures 2H, 2I, S4A, and S4B). To further support these observations, we analyzed previously published spatial transcriptomic datasets^37^ using gene set variation analysis (GSVA)-based microglial activation scores at distances of 100 and 500 μm from the injury site. This revealed a significant enrichment of activated microglial signatures in regions proximal to the implant, consistent with the localized increase in microglial density observed histologically (Figure 2H, inset).

### Coordinated morphometric programs reveal spatially organized microglial remodeling after implantation

**Figure 3:**
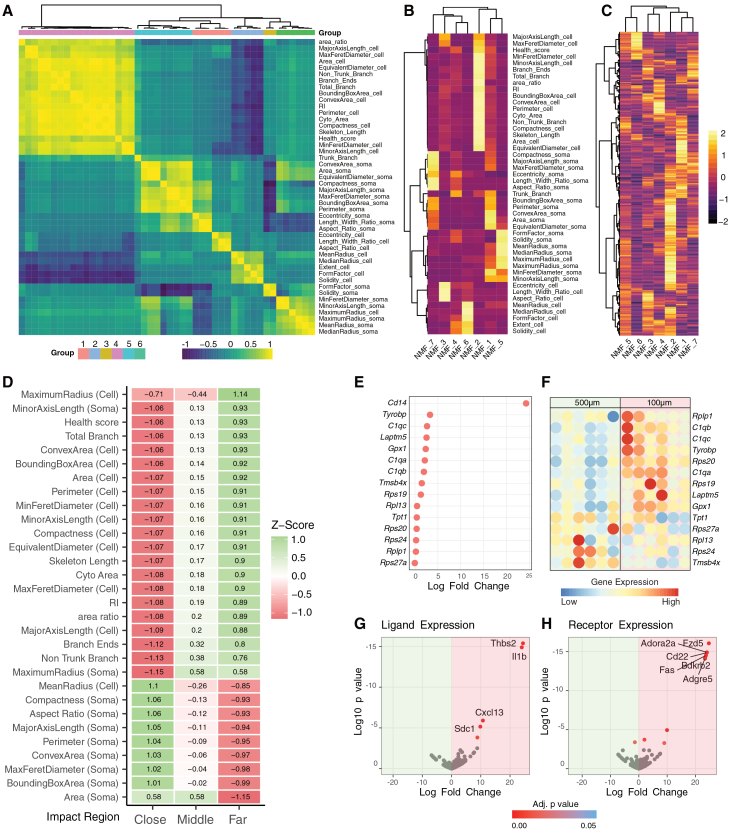
MicroFace reveals relationships among microglial morphometric parameters and spatial extent in microglia morphology and transcriptional signatures (A) Correlation structure of morphometric features. Pairwise correlation matrix of the top 48 hierarchical clustering identified six major groups of correlated features, indicating that several parameters capture related aspects of microglial morphology (*n* = 279,510 cells). (B) Non-negative matrix factorization (NMF) clustering of morphological features to identify groups of features that vary together. Seven programs were detected, many of which overlap with the correlated feature groups identified in (A), indicating consistent relationships among morphological descriptors. (C) NMF clustering of individual microglial cells. (D) Spatial differences in morphological features. *Z* score normalized values of selected morphological parameters are shown across three spatial regions relative to the implant site: close, middle, and far, highlighting systematic differences in microglial morphology depending on distance from the injury (*n* = 279,510 cells). (E) Transcriptomic comparison between samples obtained from close to the implant site to far away from implant site reveals significant upregulation of genes associated with microglial activation. Cd14 exhibits significant upregulation in samples obtained <100 μm from implantation to >500 μm from implantation site. (F) Heatmap of activated microglial gene expression at 1 week post implantation at different implantation distances. (G) Volcano plot of ligand expression in samples close to injury vs. far. (H) Volcano plot of receptor expression in samples close to injury vs. far. Points are colored by adjusted *p* value (BH correction), with limits set to 0.0 and 0.05 (note: data shown in E–H were obtained from previously published work Thompson et al.^37^).

To better understand correlations among morphological parameters, we carried out correlation analysis combined with hierarchical clustering across all extracted features. This revealed six distinct modules composed of highly correlated parameters (Figure 3A). These modules corresponded to different structural aspects of microglial morphology. Parameters in groups 4 and 2 primarily represented features derived from the entire cell, whereas groups 1, 3, and 5 captured soma-associated characteristics. The moderate correlation observed between soma-related and whole-cell parameters suggests that morphological changes following injury involve coordinated structural remodeling of both the soma and cellular processes rather than independent alterations. To further identify sets of morphological features that tend to vary together, we applied non-negative matrix factorization (NMF). Based on the cophenetic coefficient, seven NMF programs were identified, using optimal rank (Figure S5A). Mapping these programs across the morphometric features revealed distinct groups of parameters contributing to each program (Figure 3B). Specifically, NMF programs 2, 3, and 6 were largely associated with whole-cell morphological features, whereas programs 1, 5, and 7 were enriched for soma-related parameters. Program 4 predominantly captured branching and process-associated characteristics. When cells were projected onto these programs, similar feature groupings were observed across the dataset (Figure 3C). The convergence between hierarchical clustering and NMF indicates that microglial morphology can be described by coordinated structural programs.

We then examined whether these morphological programs exhibited spatial differences relative to the implant site. Microglia were assigned to three spatial regions based on their distance from the injury site: close, middle, and far regions. Several morphological parameters displayed distinct spatial patterns depending on proximity to the injury (Figure 3D). Standardized *Z* score computation across regions revealed that soma-related parameters like perimeter, compactness, mean radius, aspect ratio, and convex area were elevated in cells located near the injury site. In contrast, parameters describing overall cell size and branching complexity were reduced in this region. Conversely, in distal regions, cellular and branching features like cell area, convex cell area, ramification index (RI), number of branch endpoints, skeleton length, total branch, and cell diameter showed higher relative values. The intermediate region generally displayed values close to zero, indicating a gradual transition between these two morphological states. Together, these results indicate that microglial morphology varies systematically with distance from the injury site, consistent with a spatial gradient of cellular remodeling. To complement these morphological observations, we explored previously published transcriptomic datasets^37^ obtained from tissue within 100 μm of the implant site at 1 WPI. These data showed enrichment of genes associated with activated microglia, including *Cd14*, *Tyrobp*, and complement components *C1qa*, *C1qb*, and *C1qc* (Figures 3E and 3F). At later time points (6 WPI), the enrichment of these signatures near the injury site persisted, suggesting continued immune activity in regions proximal to the implant (Figure 2H, inset). Analysis of ligand and receptor expression further suggested active inflammatory and tissue remodeling processes in the injury-adjacent region. For example, increased *Il1b* and *Cxcl13* expression is consistent with inflammatory signaling and immune cell recruitment. Additional molecules such as *Thbs2* and *Sdc1*, which are associated with extracellular matrix remodeling and angiogenesis, were also enriched. The combined expression patterns of *Il1b*, *Adora2*, *Bdkrb2*, and *Fas* suggest a mixture of pro-inflammatory signaling and regulatory pathways that may contribute to the resolution of inflammation. Changes in molecules involved in cell adhesion and migration such as *Nectin2*, *Adgre5*, and *Sdc1* further indicate remodeling of the local cellular environment. Collectively, these molecular signatures are consistent with coordinated immune signaling and tissue remodeling in the vicinity of the implant (Figures 3G and 3H). Together, the integration of morphometric and transcriptomic analyses indicates that microglial responses to implantation are spatially structured, with pronounced morphological remodeling and immune signaling occurring near the injury site.

### High-dimensional clustering reveals spatiotemporal morphological heterogeneity in microglia

**Figure 4:**
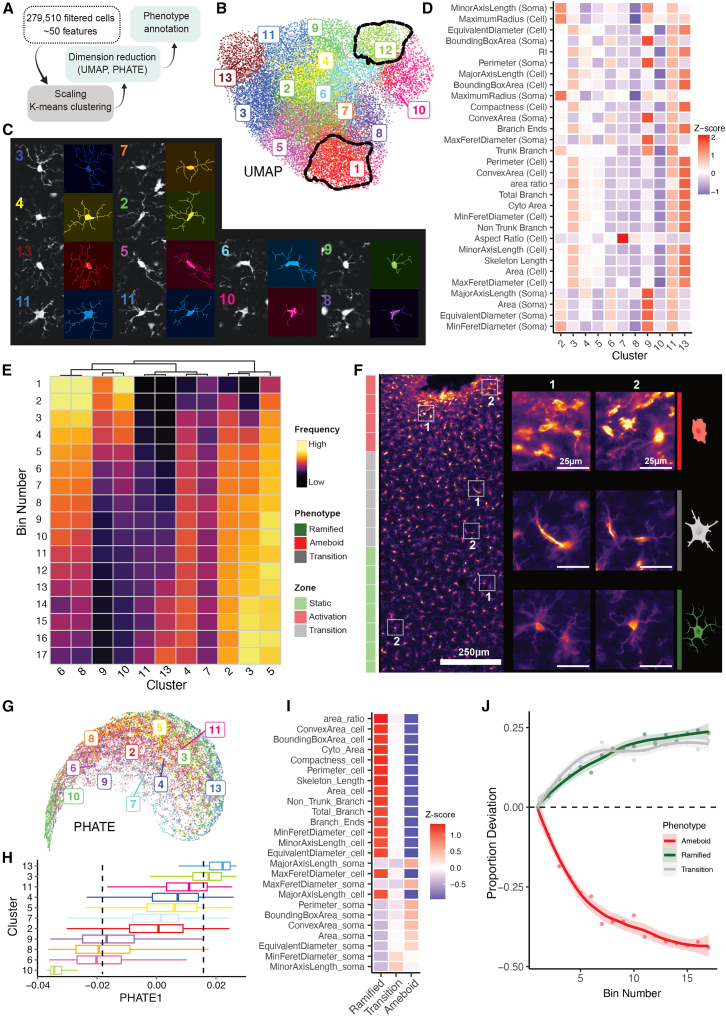
MicroFace reveals spatial dominance of distinct microglial morphotypes around the injury site (A) Overview of the morphotype identification workflow. Microglial cells (*n* = 279,510) reconstructed using the MicroFace pipeline were first filtered for quality control and then subjected to feature scaling, dimensionality reduction, and unsupervised clustering to identify groups of cells with similar morphological profiles. (B) UMAP visualization of single-cell morphological clustering. Dimensionality reduction of all analyzed cells revealed 13 distinct clusters representing different morphotypes. (Note: clusters 1 and 12 [outlined with a dotted black line] contained objects identified as segmentation artifacts or false detections upon manual inspection and were excluded from subsequent analyses.). (C) Representative examples of morphotypes. For each cluster, representative raw IBA1 immunofluorescence images (left) and the corresponding MicroFace segmentation outputs (right) are shown to illustrate the characteristic morphology captured by the clustering analysis. (D) Morphological feature profiles across clusters. Heatmap showing *Z* score normalized values of morphometric parameters across the identified clusters. (E) Spatial distribution of morphotypes across injury-centered bins. Heatmap showing the frequency of each cluster across spatial bins extending from the implant site (bin 1) to distal regions (bin 17). (F) IBA1 images showing microglia located near the implant site and in distal regions. Insets highlight representative cells illustrating morphotypes enriched in different spatial zones. (G) PHATE trajectory analysis. (H) PHATE trajectory plot illustrating the distribution of morphotype clusters along a continuous morphological progression (PHATE 1). Each boxplot represents the distribution of cells from a given cluster along the trajectory, suggesting a continuum of morphological states. (I) Morphological phenotype signatures. (J) Spatial changes in phenotype proportions. Proportions were calculated by normalizing the number of cells assigned to each phenotype within a given bin relative to the total number of cells in that bin.

To identify distinct microglial morphotypes present within the dataset, we implemented a classical data-driven analysis workflow. Morphological features extracted from 279,510 cells were first filtered for outliers using a winsorization approach and subsequently scaled prior to dimensionality reduction. Principal-component analysis (PCA) was applied for feature reduction, followed by visualization using uniform manifold approximation and projection (UMAP) and potential of heat-diffusion for affinity-based transition embedding (PHATE) embeddings. Cells were then grouped using *k*-means clustering (k = 13) to identify populations with distinct morphological characteristics (Figures 4A, 4B, and S5B). To visualize the morphological characteristics captured by the clusters, representative cells from each cluster were traced back to the original IF images and compared with their corresponding MicroFace segmentation outputs (Figure 4C). Manual inspection revealed that cluster 1 and 12 primarily consisted of segmentation artifacts or out-of-focus objects; therefore, this cluster was excluded from subsequent analyses (Figure S5C). To characterize the morphological properties of each cluster, we examined *Z* score-normalized feature values across clusters (Figure 4D). Several clusters (3, 11, and 13) showed elevated values for cellular and branching parameters, reflecting highly ramified morphologies. In contrast, clusters 2, 6, 9, and 10 displayed higher values for soma-related parameters, corresponding to more compact cellular morphologies. The remaining clusters exhibited intermediate feature profiles, suggesting morphologies positioned between these two extremes. Analysis of cellular frequency occurrence across concentric bins revealed clear spatial enrichment patterns (Figure 4E). Clusters associated with highly ramified morphologies (3 and 13) were predominantly enriched in distal regions farther from the injury site (bins 9–17). In contrast, clusters characterized by compact soma-dominated morphologies (6, 8, and 10) were enriched in regions closest to the implant (bins 1–5). Several clusters displaying intermediate morphological features (2, 5, 7, and 11) were most frequently observed in the intermediate spatial region. Based on these spatial patterns, the morphotypes could be broadly grouped into three spatial zones. Cells located closest to the injury site primarily exhibited compact morphologies with reduced branching complexity, corresponding to an activation zone (bins 1–5). Intermediate regions were enriched for cells with mixed morphological features, defining a transition zone (bins 5–10). Distal regions were dominated by highly ramified cells with complex branching structures, representing a homeostatic zone (bins 10–16) (Figures 4E and 4F).

To explore relationships among clusters, we visualized the cells using PHATE, which preserves potential nonlinear trajectories within high-dimensional data. This revealed a continuous morphological progression spanning highly ramified cells to compact morphologies, with intermediate morphotypes occupying positions along this continuum (Figure 4G). When the first PHATE dimension was examined across clusters, a distribution consistent with the previously defined spatial zones was observed (Figures 4H–4E), suggesting that the identified clusters reflect a gradual spectrum of morphological states rather than strictly discrete populations. Clusters were subsequently grouped into three broader morphological phenotypes: ramified, amoeboid, and transitional. Examination of feature profiles confirms that ramified phenotypes exhibited higher values for cellular and branching parameters, whereas amoeboid phenotypes showed elevated soma-related features (Figure 4I). Transitional phenotypes displayed intermediate feature values between these two states. Finally, we quantified the spatial distribution of these phenotypes across injury-centered bins. Ramified microglia were progressively enriched in distal regions away from the implant site, whereas amoeboid cells were most abundant near the injury and decreased in proportion with increasing distance (Figure 4J). Transitional microglia were detected across all spatial bins but were most prevalent within intermediate regions (Figure 4J). To further assess the robustness of the analysis, we examined dataset-level metadata (Figure S6; Table S1). On average, MicroFace successfully segmented approximately 4,131 microglia per animal across a total of 63 animals (Figure S6A; Table S2). When cluster proportions were evaluated for each individual animal, we observed highly consistent distributions of clusters across animals (Figure S6B). Similarly, the distribution of broader morphological phenotypes remained stable across animals and time points (Figure S6C), supporting the reproducibility of segmentation and clustering. Together, these findings reveal a spatial gradient of microglial morphotypes surrounding the implant site, with distinct morphological states distributed across injury-associated microenvironments.

### Microglial morphotypes exhibit oscillating temporal dynamics during injury response

To investigate temporal changes in morphology following chronic implantation, the cellular distribution was determined by calculating the proportion of cells assigned to each cluster and phenotype across all time points (Figures S7A and S7B). We observed clear temporal shifts in cluster as well as phenotype composition. Clusters associated with more ramified phenotype (3 and 13) showed a marked decrease shortly after implantation (Figures S7A and S7B). The combined proportion decreased from approximately 0.4 at 0 WPI to ∼0.1 at 1 WPI, followed by a gradual recovery at later time points (2, 8, and 18 WPI). Conversely, clusters representing amoeboid morphologies (8–10) showed the opposite trend. These clusters increased more than 2-fold after implantation, rising from ∼0.2 at 0 WPI to ∼0.47 at 1 WPI. After this peak, their abundance progressively declined across later time points (Figures S7A and S7B). Together, these opposing trends create a wave-like temporal pattern in which ramified morphotypes transiently decrease while amoeboid morphotypes increase during the early inflammatory phase. Notably, the transition around 2 WPI appears to represent a key inflection point in the tissue response. At this stage, the early inflammatory response begins to subside, and the system shifts toward a more reparative state. Consistent with these observations, analysis of morphometric feature *Z* scores across time revealed coordinated temporal trends (Figures S7C and S7D). Cellular and branching-related parameters decreased at 1 WPI but gradually increased at later stages (2, 8, and 18 WPI), whereas somatic parameters showed the opposite pattern, peaking at 1 WPI before declining thereafter. Interestingly, many feature *Z* scores approached near-baseline values around 2 WPI, further supporting the concept that this time point represents a transition phase between the acute inflammatory response and subsequent tissue recovery. Additionally, we examined whether the observed temporal trends were influenced by differences in probe dimensions. To this end, we compared morphotype distributions across implants with varying probe thicknesses. We observed no major differences in cluster proportions or temporal cluster dynamics when comparing across probe sizes (Figures S8A and S8B). Similarly, analysis of morphometric parameter trends revealed largely consistent patterns across all probe thickness conditions (Figures S8C and S8D). Most parameters followed comparable temporal trajectories irrespective of electrode size. Cellular parameters exhibited slightly elevated *Z* score values in the 16 μm probe condition; however, these differences were minor and are consistent with the overall temporal patterns observed across the dataset. Together, these findings indicate that the oscillating temporal dynamics of microglial morphotypes following implantation are largely conserved across probe dimensions, suggesting that the observed morphological transitions primarily reflect the intrinsic tissue response to injury rather than differences in electrode properties.

### Rod-like and reactive transitional microglia represent distinct intermediate states during injury response

**Figure 5:**
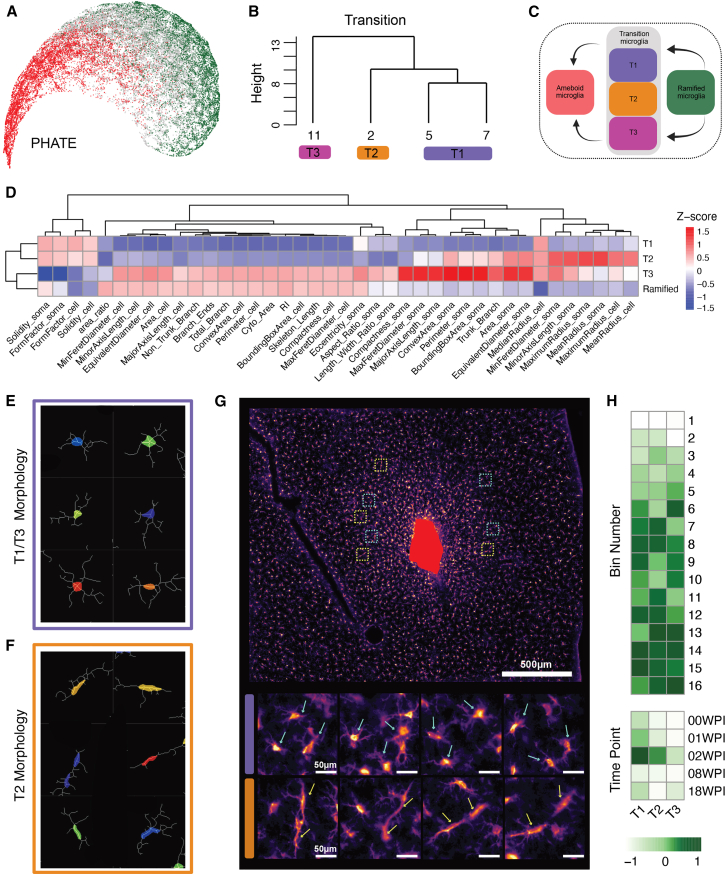
Transitional microglial phenotypes consist of distinct intermediate morphotypes (A) PHATE plot colored according to phenotype classification: amoeboid (red), transitional (gray), and ramified (green), illustrating a continuum of morphological states (*n* = 279,510 cells). (B) Dendrogram of clusters belonging to the transitional phenotype (*n* = 110,931 cells). The dendrogram reveals two major subgroups of transitional morphotypes. Clusters 5 and 7 form group T1, while cluster 2 forms group T2 and cluster 11 forms the T3 group. (C) Schematic illustrating the relationship between major microglial phenotypes. Ramified microglia transition toward amoeboid states through intermediate morphologies, which are further subdivided into multiple transitional groups. (D) Morphological enrichment analysis comparing parameters between T1–T3 and ramified morphotypes (*n* = 110,931 cells). The plot highlights morphometric features that distinguish the transitional groups. (E and F) Representative morphologies of transitional microglia. Example segmented microglia illustrating characteristic morphologies of T1/T3 (E) and T2 (F) transitional subtypes. (G) Representative IBA1 immunofluorescence image showing the spatial context of transitional morphotypes relative to the implant site. The injury location is indicated in red, and insets highlight examples of T1/T3 and T2 microglia detected near the lesion. (H) Distribution of transitional morphotypes across spatial bins and time points (*n* = 110,931 cells). The upper heatmap shows the proportion of T1–T3 cells across spatial bins extending from the implant site to distal regions. The lower heatmap shows the proportion of cells across different post-implantation time points.

To better understand the transitional characteristics of the microglial phenotypes, we projected the phenotypic classifications onto PHATE, which revealed a continuous trajectory describing the morphological progression of microglia (Figure 5A). Within this trajectory, cells with the transitional phenotype occupied intermediate positions between the ramified and amoeboid states. To further resolve the heterogeneity within this population, we performed hierarchical clustering on clusters previously classified as transitional. This analysis revealed three major branches: T1, represented by clusters 5 and 7, and T2 and T3, represented by clusters 2 and 11, respectively (Figure 5B). These findings suggest that the transition between ramified and amoeboid microglial states may occur through multiple distinct morphological pathways (Figures 5B and 5C). Visualization of the PHATE1 vs. PHATE3 dimensions further highlighted differences in the spatial density and distribution of T1–T3 cells, supporting the presence of multiple transitional programs (Figures S9A and S9B).

We then performed feature enrichment analysis to characterize the morphological differences between T1–T3 populations in comparison to the ramified population (Figure 5D). Starting with the ramified cells, we observed that the T3 population showed a highly similar enrichment of parameters, closely resembling ramified morphologies, but with additional upregulation of several somatic features. This suggests that T3 cells are closely related to the ramified state while exhibiting subtle somatic changes indicative of partial activation (Figures 5D and 5E). Consistently, T3 clusters together with ramified cells in the hierarchical analysis. In contrast, T2 displayed a distinct feature profile. Several somatic parameters, including soma area, maximum Feret diameter, major axis length, cell diameter, perimeter, and RI, were positively enriched, indicating a population with pronounced somatic elongation (Figure 5D). Additionally, T2 cells showed an increased number of trunk branches but a reduction in non-trunk branches compared with T1 (Figure 5D). These features indicate that T2 cells are characterized by elongated somas and dominant primary processes, morphological characteristics commonly associated with rod-like microglia^38^^,^^39^ (Figures 5D–5F). In contrast, T1 exhibited minimal enrichment of somatic parameters and retained a more balanced branching profile. Together, these findings suggest that T3 represents a transitional state closely aligned with the ramified phenotype, also supported by its density distribution in PHATE space near ramified cells (Figure S9B). T2 corresponds to a more distinct rod-like microglial state, while T1 occupies an intermediate position connecting T3 and T2. Notably, PHATE density further indicates that T1 cells extend toward amoeboid populations, suggesting a trajectory toward more reactive states (Figure S9B).

Spatial analysis revealed that rod-like microglia (T2) and T1 were located closer to the injury site (bins 1–7), whereas T3 cells were enriched slightly farther away, with higher representation in more distal regions (Figure 5H). However, no major differences were observed in the overall spatial distribution among the three transitional populations. Temporal analysis showed that rod-like microglia (T2) were most prevalent during the early post-implantation phase, particularly at 1–2 WPI, with a peak at 2 WPI. T1 cells also showed their highest occurrence at 2 WPI, while remaining present across other time points. In contrast, T3 cells were more prominent at later stages, particularly at 2 and 18 WPI, suggesting an association with more homeostatic phases of the tissue response. Notably, this temporal enrichment coincides with the transition point in tissue response identified in the previous analysis, where the microenvironment shifts from an acute inflammatory state toward a more reparative, quiescent phase. It remains undetermined whether individual rod-like cells transition into other morphological states, undergo apoptosis, or are replaced by newly generated or infiltrating cells.

### Exploratory transcriptomic analysis identifies a candidate molecular signature spatially coincident with rod-like microglia

**Figure 6:**
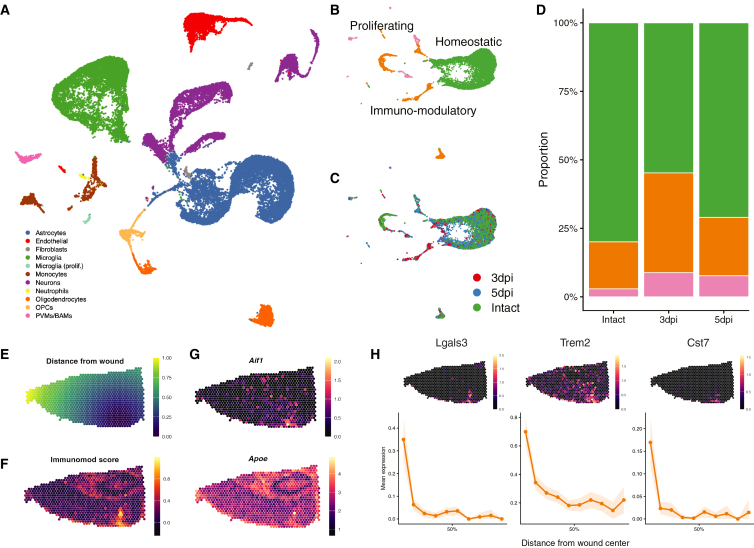
Transcriptomic identification and spatial organization of injury-responsive microglial states (A) UMAP projection of 33,357 cells from the mouse cortical stab wound dataset (GSE226211) following quality control (QC), doublet removal, and RPCA-based integration, colored by major cell classes. Microglia were subsequently isolated using canonical markers (Hexb, Tmem119, P2ry12, Cx3cr1, and Csf1r) with exclusion of non-microglial lineages. (B) UMAP of the 9,589 microglia, annotated into three transcriptional states based on module scoring: homeostatic, immunomodulatory (Immunomod), and proliferating. (C) Distribution of microglial subtypes across conditions (intact, 3 dpi, and 5 dpi), showing expansion of the Immunomod population after injury. (D) Quantification of subtype proportions across time points. The Immunomod subtype increases from ∼5% in intact tissue to ∼40% at 3 dpi, followed by partial resolution at 5 dpi. (E) Spatial map of normalized distance from the wound center in Visium data (3 dpi), used to define radial gradients. (F) Spatial distribution of Immunomod module scores (left) and Apoe expression (right). Both are enriched proximal to the wound, indicating activation within the resident microglial compartment. (G) Spatial expression of Aif1 (IBA1), showing enrichment near the injury site and confirming accumulation of microglia/macrophage-lineage cells at the wound. (H) Spatial expression (top) and radial quantification (bottom) of immunomodulatory microglia markers (Lgals3, Trem2, and Cst7), demonstrating strong proximal enrichment with decay as a function of distance from the wound center.

The morphometric analyses described earlier identified spatially and temporally organized microglial morphotypes following implantation, including a rod-like transitional state (T2) enriched at intermediate distances and peaking at 1–2 WPI. However, the molecular programs underlying these morphological states remained undefined. To address this, we reanalyzed publicly available single-cell RNA sequencing and Visium spatial transcriptomics data from a complementary brain injury model^40^ (mouse cortical stab wound; GSE226211, Koupourtidou et al.) to identify transcriptomic correlates of the morphological states observed in our rat implantation dataset. After quality control, doublet removal, and reciprocal-PCA (RPCA)-based batch integration, 33,357 cells were recovered across three conditions (intact, 3 days post-infection [dpi], and 5 dpi). Broad cell-type annotation revealed the expected diversity of neural and non-neural populations (Figures 6A and S10A**–**S10C). Cell-level gating using canonical microglial markers (*Hexb*, *Tmem119*, *P2ry12*, *Cx3cr1*, and *Csf1r*) with exclusion of monocyte, astrocyte, endothelial, and perivascular macrophage signatures yielded 9,589 microglia (Figure 6B). Module score-based annotation resolved three transcriptionally distinct microglial subtypes: homeostatic, immuno-modulatory, and proliferating (Figures 6B and S10D). The immuno-modulatory subtype was characterized by upregulation of disease-associated microglial (DAM) markers including *Trem2*, *Lgals3*, *Apoe*, and *Cst7*, alongside downregulation of homeostatic markers (*Tmem119*, *P2ry12*, and *Hexb*), consistent with a shift away from surveillance toward phagocytic and lipid metabolic programs (Figures 6G and 6H). Temporal analysis revealed dynamic changes in subtype composition following injury. The immuno-modulatory population expanded from a minor fraction in intact tissue (∼5%) to a dominant component at 3 dpi (∼40%), before partially resolving at 5 dpi (Figures 6C and 6D). This transient expansion mirrors the temporal dynamics of the rod-like morphotype (T2) observed in the rat implantation model, supporting the interpretation of both as injury-responsive intermediate states.

To assess spatial organization, we analyzed Visium data from the 3-dpi condition using section-aware wound center detection and normalized distance mapping (Figure 6E). Immunomodulatory module scores were enriched proximal to the wound (Figure 6F, left), indicating localized activation. Importantly, both the single-cell-derived immune-modulatory marker *Apoe* (Figure 6F, right) and the pan-microglial/macrophage marker *Aif1* (Figure 6G) were also enriched in the same region. This demonstrates that the injury core is populated by activated resident microglia rather than being dominated by infiltrating peripheral macrophages. Consistent with this spatial organization, DAM-associated genes (*Lgals3*, *Trem2*, and *Cst7*) showed strong proximal enrichment (Figure 6H, top), with radial quantification confirming a steep decline in expression with increasing distance from the wound center (Figure 6H, bottom). These gradients indicate a structured activation program centered on the injury site rather than a diffuse inflammatory response.

Together, these findings provide a molecular framework for interpreting the morphological heterogeneity characterized by MicroFace. The immunomodulatory transcriptional state shares key features with the rod-like transitional morphotype: both emerge transiently after injury, occupy spatially defined zones, and represent intermediate activation states. Crucially, the co-enrichment of *Apoe* and *Aif1* at the injury site indicates that these transitions occur within the resident microglial compartment. While direct mapping between morphology and transcription across species and injury paradigms remains inferential, the convergence of temporal dynamics, spatial localization, and gene expression profiles supports the interpretation that the rod-like phenotype corresponds to a DAM-like activation program within resident microglia.

## Discussion

The conventional view of microglial “activation” following TBI or neural implantation has often been portrayed as a linear progression from a “resting,” ramified state to a monolithic “activated,” amoeboid phenotype.^23^^,^^41^ Our findings, however, support a more nuanced and context-dependent paradigm. By integrating spatial, morphological, and transcriptomic analyses across brain injury modalities, we reveal a complex continuum of microglial phenotypes, each occupying distinct spatial niches and emerging at different stages of the post-injury timeline, with microglial reactivity appearing to involve a graded, dynamic response characterized by multiple intermediate phenotypes, including reactive and rod-like states.

Although morphology alone is an imperfect surrogate for microglial function, our findings indicate that morphological diversity is accompanied by distinct gene expression patterns indicative of specialized roles. Through large-scale morphometric profiling and unsupervised clustering, we identified coordinated structural programs that capture variation in soma size, cellular extent, and branching complexity. These features revealed a spatially organized gradient of microglial morphotypes surrounding the implant site, with compact morphologies enriched near the injury interface and increasingly ramified cells located in distal regions. Regions proximal to the injury showed upregulation of canonical activation markers (*Cd14*, *Tyrobp*, and *C1q* subunits) and robust inflammatory mediators (*Il1b* and *Cxcl13*), morphologically correlating strongly with compact, amoeboid cells.^42^^,^^43^ These cells, likely primed for phagocytosis and debris clearance, contrast to the more ramified morphotypes, spatially enriched in distal zones, expressing transcriptomic signatures associated with homeostatic surveillance and synaptic maintenance.^28^^,^^44^ This interplay between morphology and transcriptomic signatures substantiates the functional relevance of the observed morphological heterogeneity.

Rod-like microglia have been documented in various neurological conditions, where they are often associated with immune-modulatory roles.^38^^,^^39^ Our results that imply that these transient rod-like Iba1-positive cells express transcripts indicative of restraint on neuroinflammation (*Apoe* and *Trem2*) suggest that this distinct morphological form embodies a specialized functional subset. However, the transient abundance of this subpopulation near the injury site is consistent with several possible interpretations: rod-like Iba1-positive cells may represent a transitional intermediate between activation states, they may constitute a functionally specialized but short-lived morphotype, or their disappearance at later time points may reflect cell death and replacement rather than morphological conversion. Witcher et al. demonstrated that rod microglia align along dendrites in the cortex following diffuse brain injury,^45^ consistent with a structural relationship to neuronal elements rather than a purely incidental morphology. This abundance of the subpopulation near the injury site and its partial overlap with transient phenotypes underscore that the microglial response is not a homogeneous, unidirectional shift. Microglia navigate multiple potential “routes” toward an activated state, passing through at least two distinct transient landscapes, one resulting in a more reactive phenotype and another in rod-like phenotype aligned with immunomodulatory and reparative functions. This population could be of therapeutic interest, since the early and intermediate stages of microglial activation are precisely when critical interventions can shift the trajectory of recovery toward tissue homeostasis, exerting lasting effects on synaptic pruning, vascular remodeling, and neuronal survival,^46^^,^^47^ which could improve outcomes in TBI and enhance the integration and chronic stability of neural implants.^48^

In conclusion, this work highlights a spatially and temporally stratified repertoire of microglial phenotypes where microglia are not passive, uniform responders but agile, heterogeneous players whose morphology and molecular signatures reflect finely tuned adaptations to local injury. Our findings are critical for developing refined models of neuroinflammation and, ultimately, innovative therapeutic approaches aimed at directing microglial responses for optimal recovery and device integration.

### Limitations of the study

Several limitations should be considered when interpreting the results of this study. First, the IF images analyzed here were acquired as single optical sections rather than full z stack volumes. As a result, microglial processes extending outside the focal plane may not be fully captured, and partially out-of-focus cells may occasionally be included during segmentation, which was addressed by the implementation of multiple quality-control steps, including morphological filtering and unsupervised clustering, enabling identification and removal of clusters corresponding to segmentation artifacts. Although two-dimensional imaging does not fully represent the three-dimensional architecture of microglia, previous studies comparing 2D and 3D morphometric approaches have reported largely consistent outcomes.^49^ In particular, the overall patterns of microglial morphological responses to stimuli appear comparable between 2D projections and 3D reconstructions, suggesting that 2D representations can still provide biologically meaningful and interpretable morphological information.^49^ Finally, while morphometric analysis provides valuable insights into microglial structural states, morphology alone cannot be used to fully define functional microglial phenotypes. Although we complemented our findings with publicly available transcriptomic datasets, these data originate from independent experiments. Future studies integrating simultaneous morphological and molecular profiling will therefore be important for establishing stronger links between microglial structural features and their functional states.

Additionally, all animals received the non-steroidal anti-inflammatory drug (NSAID) carprofen as post-surgical analgesia. Carprofen inhibits cyclooxygenase activity and has been reported to reduce pro-inflammatory cytokine levels, including IL-1β and IL-6, as early as 4 h after TBI in rodent models.^50^ Because the acute samples (0 WPI) were acquired at 4 h post-implantation, carprofen administration may have partially attenuated the early inflammatory microglial response at this time point. However, as carprofen was administered uniformly across all groups, the relative morphological differences observed between time points remain internally consistent.

## Resource availability

### Lead contact

Further information and requests for resources and reagents should be directed to and will be fulfilled by the lead contact, Kevin Joseph (kevin.joseph@uniklinik-freiburg.de).

### Materials availability

This study did not generate new unique material.

### Data and code availability

• Processed morphometric datasets generated in this study are available at Zenodo.^51^ A subset of raw immunofluorescence images and corresponding segmentation masks generated using the MicroFace toolbox are also available at Zenodo.^51^ Publicly available transcriptomic datasets analyzed in this study are available from Gene Expression Omnibus (GEO) under accession numbers GSE226211 and GSE226208.
• All scripts and the MicroFace toolbox used in this study are publicly available through GitHub: https://github.com/Vatsjari/MicroFace; https://github.com/3DBMandNE-Lab/MicroFace.
• Any additional information required is available from the lead contact upon request.

## Acknowledgments

The graphical abstract and illustrations in figure panels were generated using BioRender.

## Author contributions

Conceptualization, V.D.J. and K.J.; methodology, V.D.J., K.J., and U.G.H.; investigation, K.J. and U.G.H.; software, V.D.J. (MicroFace toolbox); formal analysis, V.D.J. and K.J.; validation, S.P.; resources, V.M.R., J.B., U.G.H., and K.J.; writing – original draft, V.D.J. and K.J.; writing – review and editing, all authors.

## Declaration of interests

The authors declare no competing interests.

## STAR★Methods

### Key resources table

**Table:** 

REAGENT or RESOURCE	SOURCE	IDENTIFIER
**Software and Algorithms**		

MicroFace V1.1.0	This study	https://github.com/Vatsjari/MicroFace; https://github.com/3DBMandNE-Lab/MicroFace
Fiji (ImageJ v1.53)	Schindelin et al.	https://fiji.sc
CellProfiler v4.2	McQuin et al.	https://cellprofiler.org
R v4.2/v4.5.1	R Core Team	https://www.r-project.org
Seurat v5.4.0	Hao et al.	https://satijalab.org/seurat
ZEN (Blue edition)	Carl Zeiss Microscopy	https://www.zeiss.com/microscopy/en/products/software/zeiss-zen.html

**Biological Samples**		

Sprague–Dawley rats	This study	Female, 8–10 weeks
Cortical implantation model	This study	Flexible neural probe implantation

**Antibodies**		

Iba1	Wako	1:1000 (Cat# 019-19741; RRID: AB_839504)
GFAP	Dako	1:500 (Cat# Z0334; RRID: AB_10013382)
NeuN	Synaptic Systems	1:500 (Cat# 266004)
Secondary antibodies	Dianova	Cy2/Cy3/Cy5 (1:100)
Alexa Fluor 633	Thermo Fisher	1:100
Chemicals/Reagents		
Carprofen	Vet supplier	5 mg/kg
Paraformaldehyde	Standard supplier	4% PBS
Triton X-100	Sigma-Aldrich	0.25%
DAPI	Thermo Fisher	1:10000
BSA	Sigma-Aldrich	1% solution

**Deposited Data**		

Morphometric dataset	This study	https://doi.org/10.5281/zenodo.20325902
IF raw images	This study	https://doi.org/10.5281/zenodo.20325902
Segmentation masks	This study	https://doi.org/10.5281/zenodo.20325902
scRNA-seq dataset	Koupourtidou et al.	GSE226211
stRNA-seq dataset	Koupourtidou et al.	GSE226208
Bulk RNA-seq dataset	Thompson et al.	J. Neural Eng.
Experimental Models		
Neural probe implantation	This study	Rat cortical microinjury
Flexible neural probes	IMTEK Freiburg	6–50 μm thickness

### Experimental model and study participant details

This study employed an *in vivo* rat model of localized cortical microinjury induced by implantation of flexible neural probes to investigate spatiotemporal remodeling of microglial morphology following neural interface implantation. Adult female Sprague–Dawley rats were analyzed across multiple post-implantation time points (0, 1, 2, 8, and 18 WPI). Microglial morphology was quantified from IBA1 immunofluorescence images using the MicroFace pipeline for automated single-cell segmentation and morphometric profiling. Publicly available transcriptomic datasets were additionally incorporated for comparative and integrative analyses. This study did not involve experimental human participants, primary cell cultures, or experimental cell lines.

### Method details

#### Flexible neural probe design

Flexible neural probes were fabricated on a polyimide substrate using micromachining methods described previously.^52^ The probes measured 380 μm in width and 15 mm in length and were produced with thicknesses of 6, 11, 18, or 50 μm. Each probe contained 12 recording sites (15 μm^2^) and 4 stimulation sites (50 μm^2^). These probe designs were originally developed for electrophysiological recording applications; however, in the present study the IF images were used only to study the associated tissue response. The different probe thicknesses were included to reflect the range of devices used in prior implantation studies using this platform. Probes were designed and manufactured at IMTEK (Freiburg, Germany).

#### Animal procedures

All animal experiments were conducted in accordance with the guidelines of the German Council on Animal Protection. Experimental protocols were approved by the Animal Care Committee of the University of Freiburg and authorized by the Regierungspräsidium Freiburg (approval number G15/031), in compliance with European Union Directive 2010/63/EU. Adult female Sprague–Dawley rats (8–10 weeks old, 200–250 g) were used in this study. Animals were housed under standard laboratory conditions with a 12 h light/dark cycle and free access to food and water. A total of 63 animals were used across all experimental groups. For morphological image analysis rats were assigned to the following time points: 0, 1, 2, 8, and 18 WPI (*n* = 13, 12, 16, 6, and 16 animals respectively). Only female animals were used to reduce variability associated with sex-dependent differences in immune responses that have been reported in some brain injury models. Because only female animals were included, sex-dependent differences in microglial morphology could not be evaluated in this study and should be considered a limitation of the findings.

#### Surgical procedure

Cranial trepanation was performed above the designated sampling region for both the control and implanted samples to account for the potential influence of skull trepanation on the transcriptional profile. Initial anesthetization involved a mixture of 5% isoflurane and oxygen (1–2 L/min) until the animal was securely positioned in the stereotactic frame (David Kopf, USA), after which the isoflurane concentration was reduced to 1.5%. Continuous monitoring of animal reflexes, breathing, and depth of anesthesia was maintained throughout all surgical procedures. The surgical protocol up to craniotomies followed established methodologies detailed in previous reports.^53^^,^^54^^,^^55^ Implantation coordinates were determined relative to the bregma position, with two implantation sites at AP_Bregma_ ±0 mm, ML_Electrode1_ = ML_Bregma_ +1.5 mm, and ML_Electrode2_ = ML_Bregma_ −1.5 mm, and two implantation sites at AP_Bregma_ −2.5 mm, ML_Electrode3_ = ML_Bregma_ +1.5 mm, and ML_Electrode4_ = ML_Bregma_ −1.5 mm. These locations correspond to cortical regions spanning the motor and somatosensory cortex. Micro-craniotomies were performed at specified coordinates, with precautions taken to minimize cortical surface heating. Sterile 0.9% NaCl (B'Braun, Germany) was used to flood the surgical zone during short bursts of craniotomy, and efforts were made to prevent contact between the drill bit and the dura or upper cortical layers. The craniotomies were rinsed with 0.9% NaCl to remove bone debris before probe insertion.

#### Probe implantation

For standardized implantation, a commercially available fiber optic cannula (CFMLC21L02, Thorlabs, USA) with a diameter of 105 μm and length of 2 mm served as the implantation shuttle and was mounted on a standard cannula holder (XCL, Thorlabs, USA). To prevent cross-contamination, each fiber-optic cannula was exclusively used for implantation in a single animal. Probes, unpacked, and rinsed in 70% ethanol followed by sterile distilled water, ensured proper wetting of the electrode sites and prevented air bubble entrapment before implantation. All the probes were trimmed to a length of 2 mm to maintain procedural uniformity. After lubrication with 0.9% NaCl, trimmed probes were positioned on the skull surface and inserted smoothly at the designated coordinates of interest. The electrodes were implanted to a depth of 2 mm, spanning all cortical layers, with the probe tip positioned at the interface between the gray and white matter.

#### Post-surgical care

Analgesics (carprofen, 5 mg/kg/day, subcutaneous) were administered to all animals in accordance with institutional veterinary guidelines, starting immediately post-surgery. Animals underwent a minimum recovery period of five days before sample collection, except for the acute group (0 WPI), which were sacrificed 4 h post-implantation. Carprofen was administered uniformly across all experimental groups and time points.

#### Immunohistochemistry

For subsequent immunocytochemistry, the rats were anesthetized and subjected to transcardial perfusion with 0.9% NaCl solution, followed by perfusion with 4% paraformaldehyde in phosphate-buffered saline (PBS, pH 7.4) at specified time points after probe implantation. The brains were then post-fixed in the same fixative overnight at 4°C and serially sectioned on a vibratome (50 μm, horizontal plane) for free-floating IF staining. Tissue sections were preincubated in 0.25% Triton X-100 and 10% normal serum in PBS for 30 min or in 1% bovine serum albumin in PBS. The sections were then incubated with primary antibodies (GFAP- 1:500, Dako; Iba1- 1:1000, Wako; NeuN- 1:500; Synaptic Systems) for 4 h at room temperature, followed by an overnight incubation at 4°C. Secondary antibodies coupled to CyTM2 (1:800), CyTM3 (1:400), CyTM5 (1:400) from Dianova, or Alexa 633 (1:100, Thermo Fisher Scientific) were applied in the dark for 2 h at room temperature. Simultaneously, all sections were counterstained with 4′,6-diamidino-2-phenylindole (DAPI, 1:10000). After rinsing, the sections were cover slipped with a fluorescence mounting medium (Dako).

#### Image acquisition

Sections were imaged using a widefield epifluorescence microscope (AxioImager, Zeiss) equipped with a Plan-Apochromat 10× objective (NA 0.45) and an AxioCam MRm digital camera. Images were acquired using Zen software (Zeiss). Imaging was performed using single optical planes to enable consistent high-throughput acquisition across large tissue areas. The final image resolution was 0.44 μm per pixel. Widefield epifluorescence imaging was selected to allow acquisition of large cortical regions containing the implantation track and surrounding tissue. Although this approach does not provide optical sectioning comparable to confocal microscopy, it enabled systematic analysis of large numbers of cells across extended tissue regions.

#### MicroFace toolbox

To enable large-scale quantitative analysis of microglial morphology, we developed MicroFace, an automated image processing pipeline that extracts morphometric features from Iba1-labeled IF images. The workflow consists of four major stages: image preprocessing, cell segmentation, skeleton reconstruction, and feature extraction (Figures 1 and S1). All image processing steps were implemented using Fiji (ImageJ v1.53) and CellProfiler (v4.2), with custom scripts written in R (v4.2) for downstream analysis.

#### Image preprocessing

Raw IF images were first subjected to preprocessing in Fiji^35^ to improve signal-to-noise ratio and ensure consistent segmentation across datasets. Brightness and contrast were automatically adjusted using the Fiji^35^ “Auto” function. Background fluorescence was removed using the rolling ball algorithm (radius = 50 pixels). To improve dynamic range, pixel saturation was limited to the top and bottom 1% intensity values.

#### Illumination correction

Nonuniform illumination can introduce bias and intensity variations, which negatively impact segmentation and quantification. Briefly, median filter (radius = 20 pixels) was used to generate a variable intensity background and that was subtracted from the original image to correct the uneven illumination cause due to variability in microglia intensity that was higher close to injury location, helping the segmentation and thresholding to be uniform across all images.

#### Object segmentation

Microglial somas and processes were segmented from illumination-corrected images using a multistep workflow implemented in CellProfiler. First, soma detection was performed on a median-filtered version of the input image to reduce local noise. Median filtering was applied prior to thresholding. Microglial somas were identified using Otsu global thresholding with the following parameters: minimum threshold = 0.1, adaptive thresholding strategy, threshold smoothing filter = 0.3488, threshold correction factor = 1.0, and adaptive window size = 50 pixels. To separate adjacent or touching somas, clump-splitting was applied using the shape-based method, with intensity-based dividing lines used to distinguish closely positioned cells. Objects touching the image borders were excluded from further analysis. To enhance microglial processes, neurite-like structures were emphasized using the tubeness filter with a smoothing scale of 1.2. Branch structures were then detected using the previously identified soma objects as seeding points. Branch propagation was performed using a propagation-based segmentation approach with a global threshold strategy. Thresholding for branch detection was performed using the minimum cross-entropy method, with smoothing scale = 2 and threshold correction factor = 0.6. The resulting soma and branch segmentations were combined to generate complete cell profiles for downstream skeleton reconstruction and morphometric feature extraction.6. A detailed step-to-step guide for the MicroFace pipeline, including module definition and representative illustrations, is provided in Figure S11A.

#### Skeletal analysis

Skeletal analysis focuses on extracting the central structure of microglia, known as their skeleton or backbone. Identifying the soma and branches led to the creation of a binary image of the branches. A connecting skeleton was generated through mathematical algorithms, preserving the topology and spatial relationships. These skeletons were quantified to obtain features such as the length, number of branches, and endpoints. Skeleton measurements were calculated from the segmented branch structures and therefore represent process architecture rather than soma size.

#### Parameters

We identified and measured over 70 morphometric features from 63 animals, accounting for >279,000 microglia and the shape and spatial arrangement of these cells. These features fell into three main domains: 1) Basic Shape Descriptors: Radius, surface area, and perimeter, 2) Skeleton Properties: Branch length, number of branches, and number of endpoints specific to the ramified nature of microglia, and 3) Localization Information: *x* and *y* coordinates of the cell and soma. The list and definitions of these parameters are provided in Figure S11B and Tables S3 and S4.

#### Concentric circles for binning

To evaluate the spatial distribution of cells around the injury site, the image was divided into concentric circles originating from the centroid of the injury. A total of 16 concentric circles were established, each with a radius equal to that of the previous circle, and a fixed increment of 140-pixel units. This increment was chosen to ensure that the outer boundary of the last circle extended just beyond the cells farthest from the site of injury. Furthermore, placement of the last circle ensured that none of the 16 sectors touched the image border. Bins were further classified based on their proximity to the implantation site: “Near” represented bins 1–4 (colored red), which were in closer proximity, while “Far” represented bins 10–16 (colored green), indicating greater distance from the implantation site.

The radial distance of each cell from the center of the injury was calculated using the following formula:

Radial distance = √((X_cell_-X_Center of Injury_))ˆ2+(Y_cell_-Y_Center of Injury_))ˆ2).

Where: X_cell_ = X-coordinate of the cell, Y_cell_ = Y-coordinate of the cell, X_Center of Injury_ = X-coordinate of injury center, Y_Center of Injury_ = Y-coordinate of injury center.

### Quantification and statistical analysis

#### Morphometric analysis

##### Data preprocessing and feature selection

Morphological data were analyzed in R. The combined dataset was partitioned into metadata and quantitative morphological features (remaining columns). Only numeric feature columns were retained for downstream analyses.

##### Outlier detection

Multivariate outliers were identified using the Mahalanobis distance, which accounts for correlations between features. Distances were calculated from the winsorized feature matrix using the global mean vector and covariance matrix. A cutoff corresponding to the 99.9th percentile of the chi-squared distribution (degrees of freedom equal to the number of features) was applied. Cells exceeding this threshold were classified as outliers and excluded from further analyses.

During clustering analysis, a cluster enriched for segmentation artifacts was identified and removed from subsequent analyses. This filtering step ensured that downstream morphometric analyses reflected biologically relevant microglial morphologies.

### Statistical analysis

All data analyses were conducted in R-studio, commencing with metadata annotation and scaling. All analyses were performed on cross-sectional datasets at each time point. Individual cells were not tracked longitudinally across time. Prior to analysis, morphometric features were scaled and filtered for outliers using a winsorization approach to minimize the influence of extreme values. To explore relationships between morphological parameters, Pearson correlation analysis followed by hierarchical clustering was performed. Dimensionality reduction was performed using PCA followed by UMAP and PHATE^56^ embedding to visualize nonlinear trajectories in morphometric space. Microglial clusters were identified using k-means^57^ clustering, with the optimal number of clusters determined using the elbow method. Heatmaps and feature visualizations were generated using the pheatmap package in R.^58^

Segmentation outputs were manually inspected for potential artifacts such as out-of-focus objects or incomplete cell detections. During clustering analysis, clusters enriched for segmentation artifacts was identified and removed from subsequent analyses. This filtering step ensured that downstream morphometric analyses reflected biologically relevant microglial morphologies.

#### Transcriptomic analysis

##### Bulk RNA-seq (rat, thompson et al., 2021)

Raw count data from Thompson et al. (J. Neural Eng., 2021) were obtained from the original authors. Count matrices were normalized using DESeq2 (v1.40) variance stabilizing transformation. Differential gene expression between samples obtained <100 μm and >500 μm from the implant site at 1 WPI was calculated using the Wald test, with Benjamini–Hochberg false discovery rate (FDR) correction (significance threshold: adjusted *p* < 0.05, |log2FC| > 0.5). Gene set enrichment was performed using fgsea (v1.26) with MSigDB hallmark and immune-related gene sets. Ligand–receptor enrichment analysis was carried out by intersecting differentially expressed genes with the curated CellChatDB v2.1 ligand–receptor database.

##### Single-cell RNA-seq (mouse, koupourtidou et al., 2024, GSE226211)

To identify transcriptomic correlates of the morphological states characterized by MicroFace, we reanalyzed publicly available single-cell RNA sequencing data from a mouse cortical stab wound model (GSE226211; Koupourtidou et al., 2024). Raw count matrices for 10 control samples (5 intact, 2 three-days post-injury [3 dpi], 3 five-days post-injury [5 dpi]) were obtained from GEO (GSE226207) and processed using Seurat v5.4.0^59^ in R v4.5.1. Cells were filtered to retain those with 200–6,000 detected genes and <10% mitochondrial reads. Predicted doublets were identified using scDblFinder v1.18^60^ run per sample with default parameters and removed (2,256 doublets; 6.3% overall), yielding *n* = 33,357 cells. Data were log-normalized, and 3,000 variable features were identified using variance-stabilizing transformation. After scaling with regression of mitochondrial percentage, PCA was performed (30 components). Batch correction across samples was performed using reciprocal PCA (RPCA) integration via Seurat v5 IntegrateLayers (k.anchor = 20). Clustering was performed on the RPCA-integrated reduction (dims 1–20) at resolution 0.5, and UMAP embeddings were computed on the same reduction. Major cell types were annotated based on canonical marker expression: microglia (*Hexb*, *Tmem119*, *P2ry12*), astrocytes (*Gfap*, *Aqp4*, *Slc1a3*), neurons (Rbfox3, Snap25, Syt1), endothelial cells (*Cldn5*, *Pecam1*), oligodendrocytes (Mog, Mbp, Plp1), OPCs (*Pdgfra*, *Olig2*), monocytes (Ly6c2, *Ccr2*), perivascular macrophages (*Lyve1*, *Cd163*), fibroblasts (*Col1a1*, *Dcn*), and neutrophils (S100a8, S100a9). Microglia were isolated using a cell-level gating strategy after RPCA integration. Cells were required to express at least 3 of 5 canonical microglial markers (*Hexb*, Tmem119, P2ry12, *Cx3cr1*, *Csf1r*), including at least one core marker (*Hexb*, *Csf1r*, or *Cx3cr1*), while expressing no more than 2 of 12 contaminant markers (*Ly6c2*, *Ccr2*, *Cldn5*, *Pecam1*, *Gfap*, *Aqp4*, *Lama1*, *Dcn*, *Lyve1*, *Cd163*, *S100a8*, *S100a9*). An additional gate captured DAM-like cells expressing ≥2 of 5 positive markers with low contaminant expression. This yielded 9,589 microglia. After dimension reduction and clustering, subtypes were assigned using curated gene sets for Homeostatic (*Tmem119*, *P2ry12*, *Hexb*, *Cx3cr1*, *Cst3*, *Sparc*, *Siglech*, *Selplg*, *Gpr34*), DAM (*Trem2*, *Tyrobp*, *Cst7*, *Lpl*, *Apoe*, *Axl*, *Itgax*, *Clec7a*, *Cd9*, *Spp1*), Immunomodulatory (*Cd83*, *Lgals3*, *Hif1a*, *Tgfb1*, *Mrc1*), Inflammatory (*Il1b*, *Tnf*, *Ccl2*, *Ccl3*, Ccl4, *Cxcl10*, *Nfkbia*, *Ptgs2*, *Cd14*, *Cd86*), and Proliferating (*Top2a*, *Mki67*, *Stmn1*, *Birc5*, *Cdk1*) signatures. Clusters were then simplified into broader subtypes. Immunomod-defining genes were identified using Wilcoxon rank-sum tests (FindMarkers, one-vs-rest, logFC threshold 0.5, min.pct 0.1). Temporal composition differences were assessed by Kruskal-Wallis tests on per-sample subtype proportions across conditions, with Benjamini-Hochberg FDR correction.

##### Spatial transcriptomics (mouse, koupourtidou et al., 2024, GSE226211)

Visium spatial transcriptomics data for the 3-dpi stab wound sample (GSM7068163, GSE226208) were obtained from GEO. Tissue positions and count matrices were loaded and filtered to in-tissue spots (*n* = 1,740). Because the 3-dpi slide contained two tissue sections, Gaussian mixture modeling (Mclust, G = 2) was applied to spot coordinates to separate sections. Wound centers were identified per section as the median coordinates of spots in the top 5^th^ percentile of mean expression of inflammatory markers (Cd14, Tyrobp, C1qa, Lyz2, Lgals3). Euclidean distances from each spot to its section-specific wound center were computed and normalized to [0, 1] within each section. To map microglial transcriptomic states onto the tissue, module scores were computed for each subtype using AddModuleScore (Seurat) on log-normalized Visium counts with the same gene sets used for scRNA-seq subtype annotation: Immunomodulatory (*Trem2*, *Lgals3*, *Apoe*, *Cst7*, *Cd83*, *Tyrobp*, *Lpl*, *Axl*, *Itgax*, *Cd9*), Homeostatic (*Tmem119*, *P2ry12*, *Hexb*, *Cx3cr1*, *Cst3*, *Sparc*, *Siglech*, *Selplg*, *Gpr34*), and Proliferating (Top2a, Mki67, Stmn1, Birc5, Cdk1). Spatial gene expression maps were generated for individual DAM-associated markers (Lgals3, Trem2, Cst7) and the pan-microglial marker Aif1 (Iba1) alongside the microglia marker *Hexb*. To quantify spatial enrichment, mean expression of DAM-associated markers was computed across 10 equidistant bins by normalized wound distance, with 95% confidence intervals estimated from per-bin standard error.

## Supplemental information

Document S1. Figures S1–S11 and Tables S2–S4

Table S1. Metadata table reporting the total number of segmented microglial cells and number of analyzed images for each animal included in the study
