# Supplementary material for: MicroFace maps microglial morphology remodeling, revealing spatial zones and bifurcated trajectories during brain microinjury recovery
Source: iScience. 2026 Jun 23;29(7):116485. doi: 10.1016/j.isci.2026.116485 (PMC13320269; doi:10.1016/j.isci.2026.116485)
Supplement: Document S1. Figures S1–S11 and Tables S2–S4 [file mmc1.pdf]

iScience, Volume *29*

Supplemental information

MicroFace maps microglial morphology remodeling, revealing spatial zones and bifurcated trajectories during brain microinjury recovery

Vatsal D. Jariwala, Shreya Ponnamma, Vidhya M. Ravi, Jürgen Beck, Ulrich G. Hofmann, and Kevin Joseph

SUPPLEMENTARY FIGURES

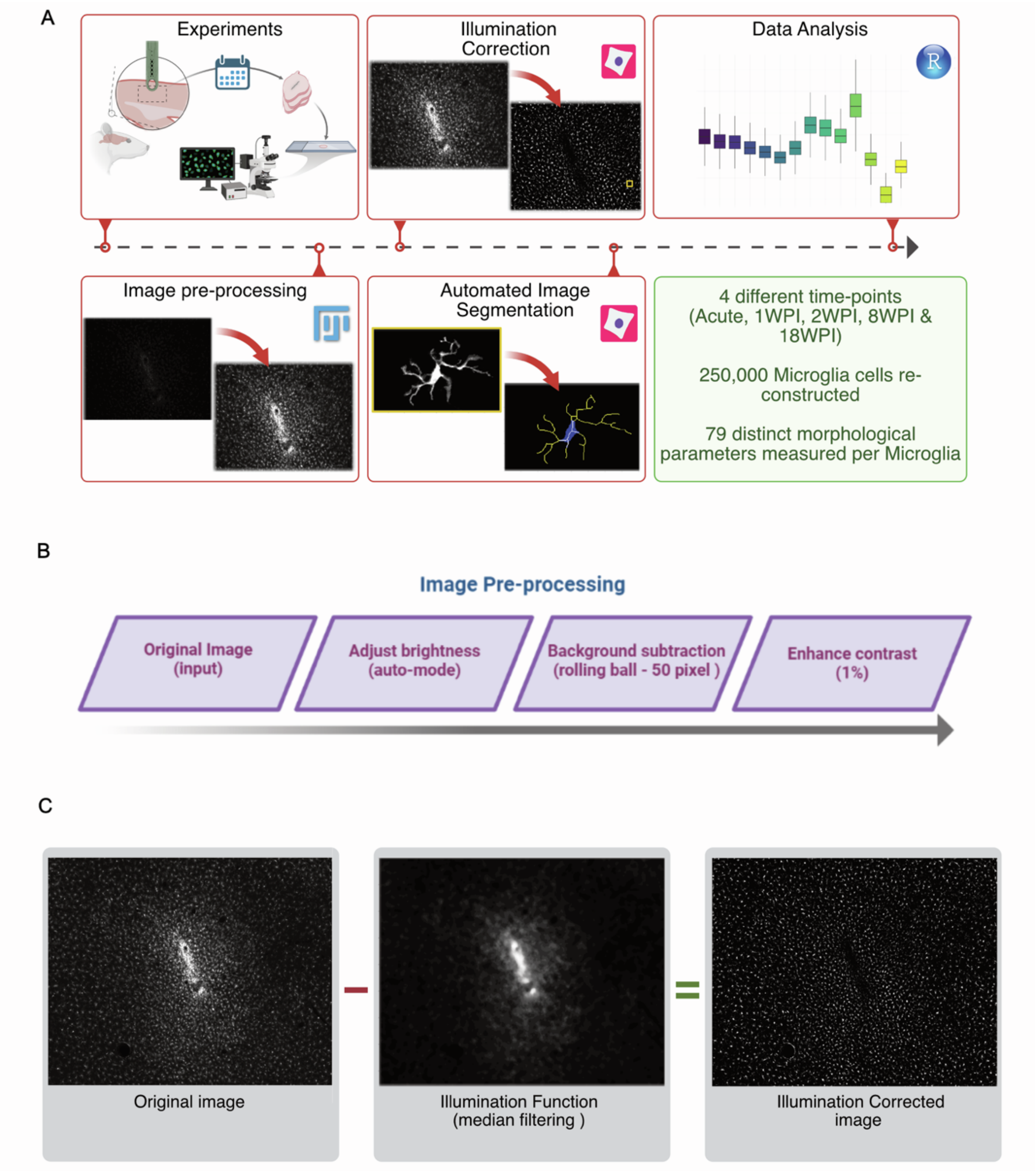

Figure S1 Schematic diagram of the workflow used to develop MicroFace.

A) The experimental procedures which were carried out previously. Step:1 Image pre-processing of the raw images to adjust brightness of the image using FIJI. Step2: Illumination correction to remove the uneven background from the image using CellProfiler pipeline. Step3: Automated image segmentation of microglia skeleton using CellProfiler pipeline. Step4: Data analysis using R-studio.

B) Image pre-processing block diagram (implemented in FIJI)

C) Iba1 stained images before and after illumination correction. 1- the original image where we can see high intensity of pixel values at the trauma site. 2- corresponding illumination function calculated using the original image. 3- subtracting the illumination function from the original image to get illumination corrected image.

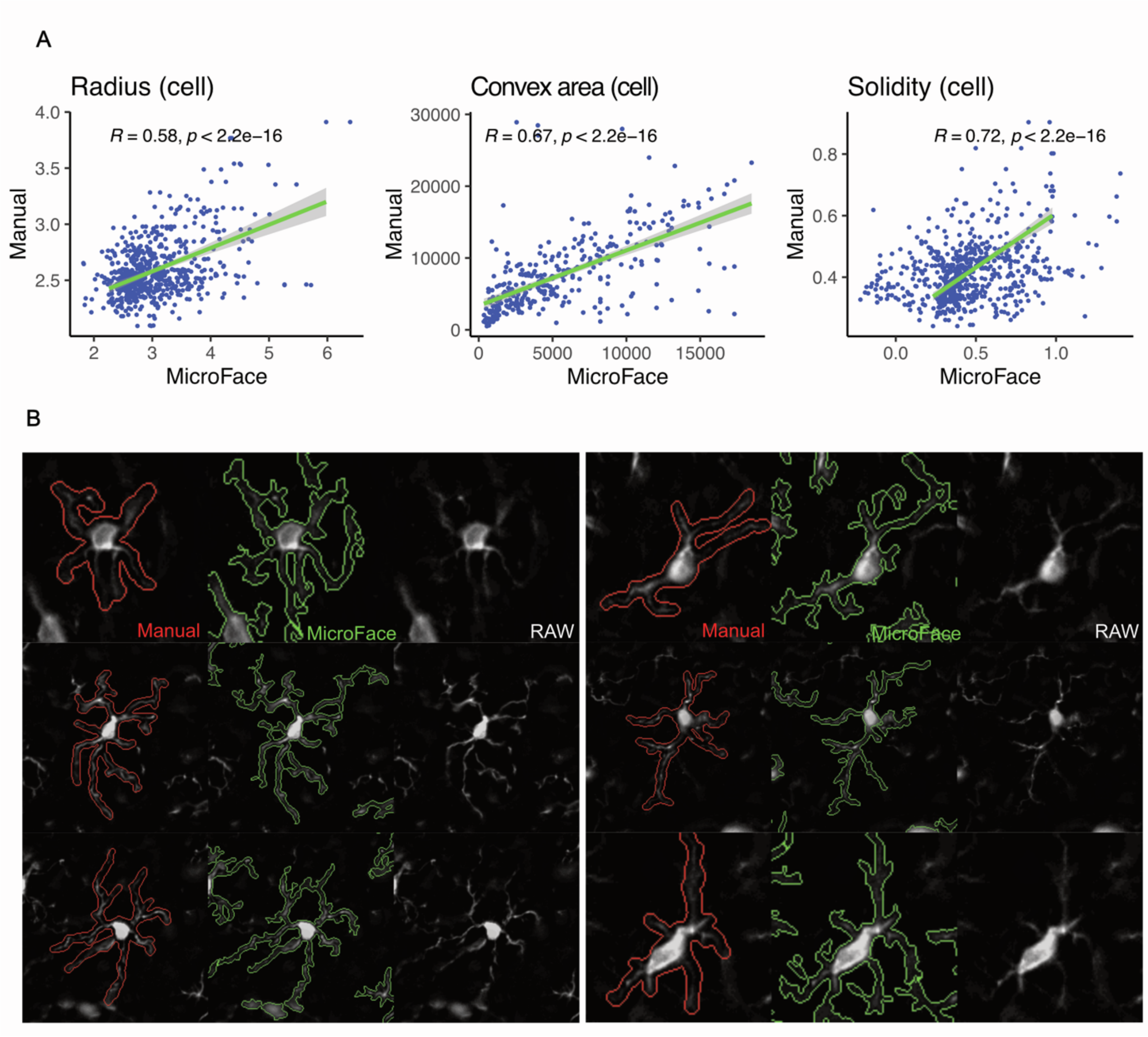

Figure S2 MicroFace validation vs manual segmentation

A) Correlation between MicroFace and manual segmentation measurements. Scatter plots compare morphological features extracted from MicroFace (x-axis) with values obtained from manual segmentation (y-axis). Cell radius (n = 326 cells, r = 0.58), convex area (r = 0.67), and solidity (r = 0.72). Each point represents a single microglial cell.

B) Examples (n=6) highlighting differences between segmentation approaches. Representative raw images are shown alongside MicroFace and manual segmentation outputs. In some cases, fine distal branches detected by MicroFace are not captured during manual tracing.

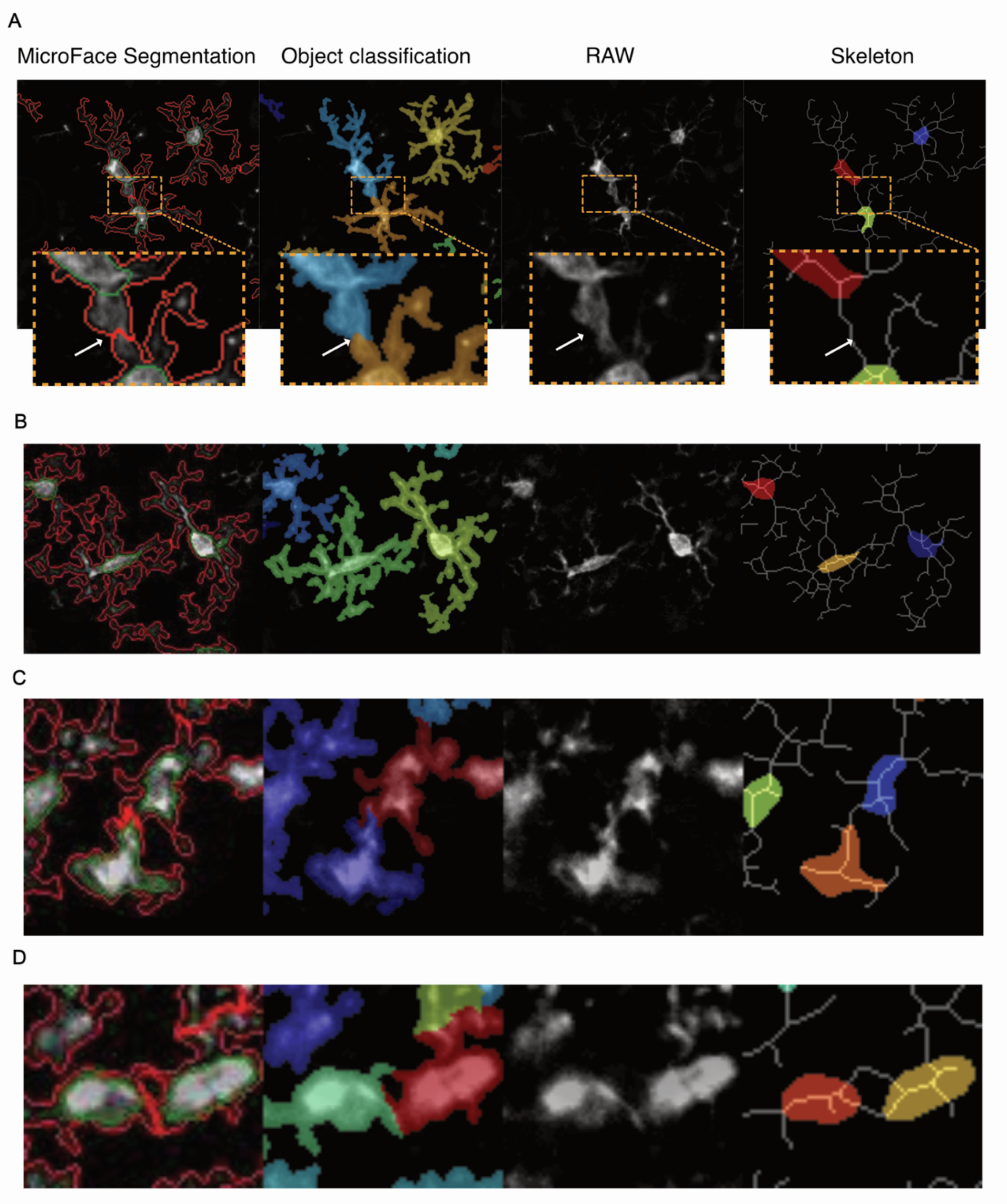

Figure S3 MicroFace accurately separates adjacent microglial processes and somas

A–B) Examples of neighboring microglial processes located in close proximity. MicroFace segmentation demonstrates separation of overlapping branches belonging to adjacent cells using intensity gradients and morphological filtering.

C–D) Representative images illustrating segmentation of closely positioned microglial somas within densely populated injury-adjacent regions

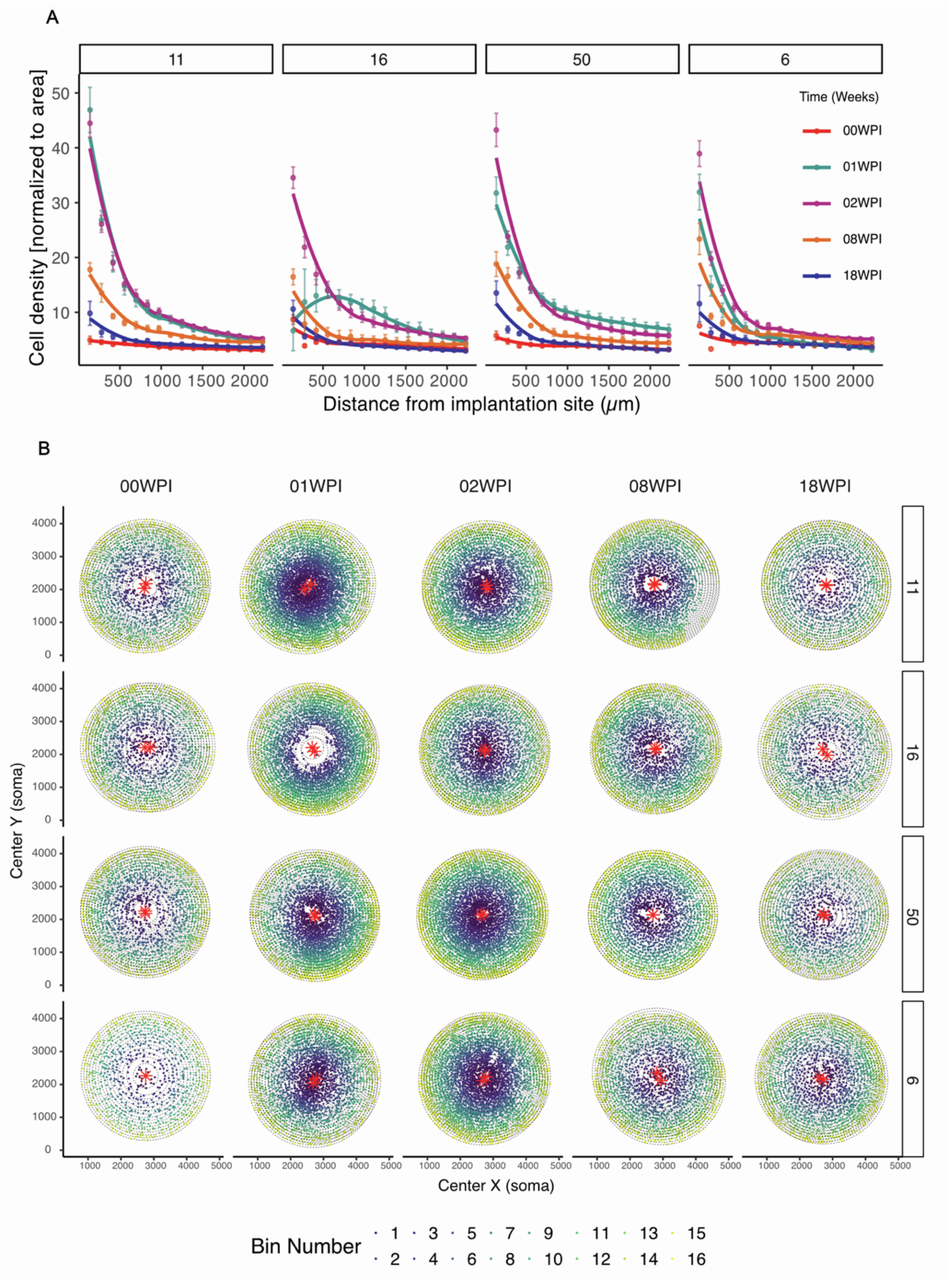

Figure S4 Spatial microglial density patterns across probe thickness conditions.

A) Quantification of microglial density across spatial bins surrounding the implant site for probes of different thicknesses.

B) Comparison of spatial distribution profiles across electrode sizes showing consistent patterns of microglial accumulation near the injury site regardless of probe thickness (n=2 per time points and electrode thickness).

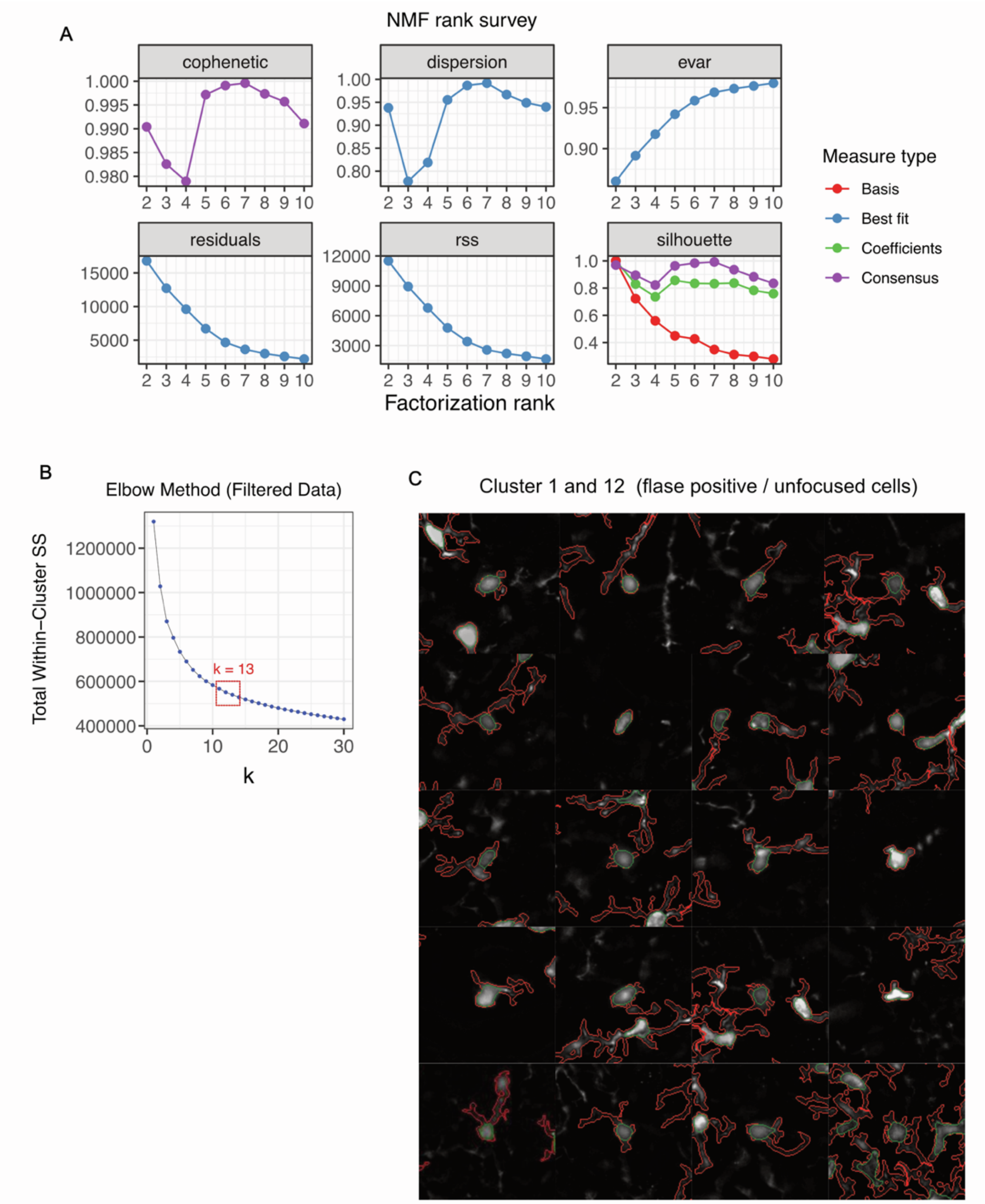

Figure S5 Identification of morphometric feature programs and cluster validation

A) Cophenetic coefficient plot used to determine the optimal rank for NMF analysis of morphological parameters.

B) Elbow method plot used to determine the optimal number of clusters during k-means clustering of microglial morphometric features.

C) Example snipets (n=16) from Cluster 1 and 12 identified as segmentation artifacts or out-of-focus detections and excluded from downstream analyses.

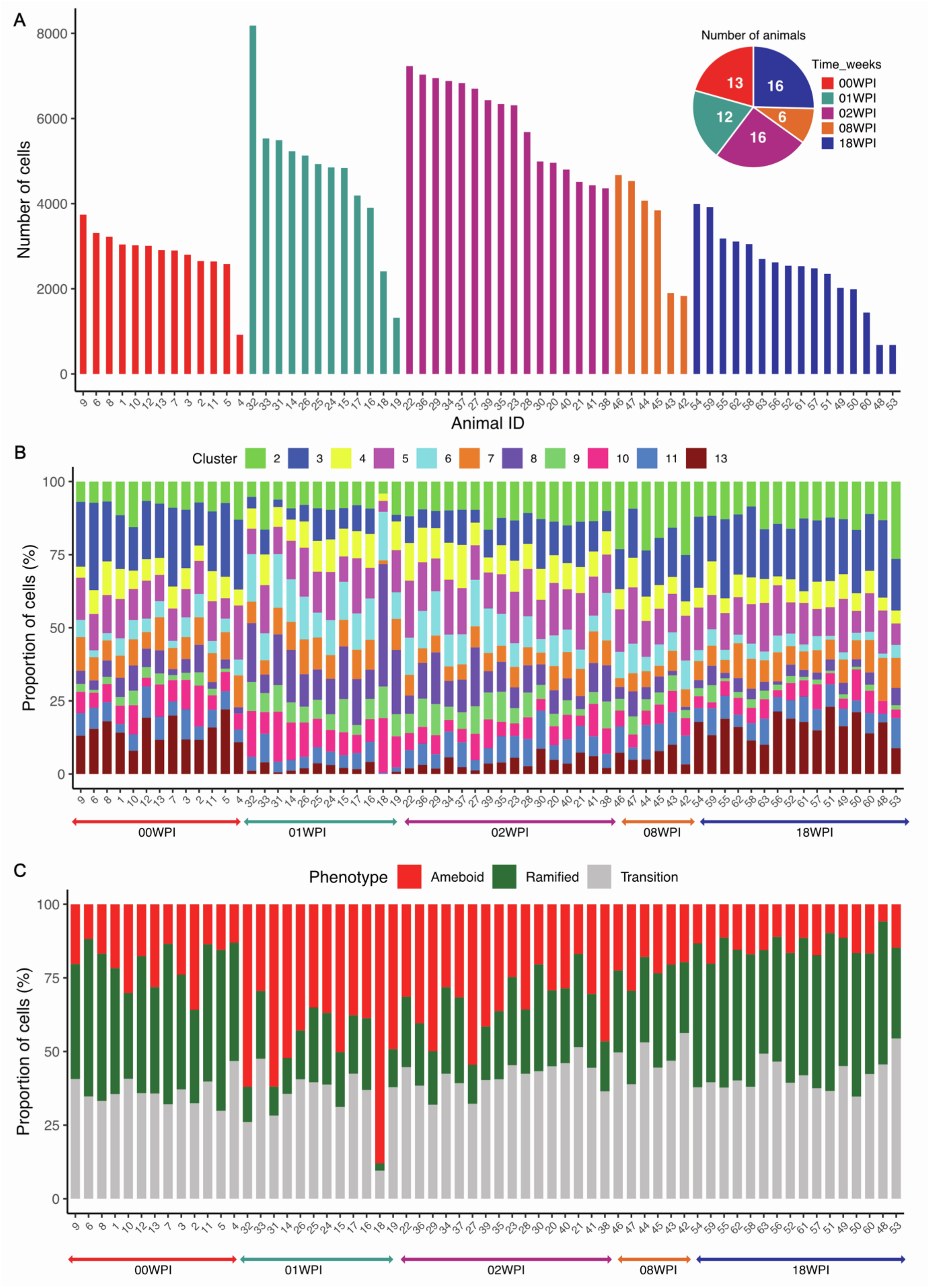

Figure S6 Metadata (QC) of segmentation across animals across all timepoints.

A) Total number of segmentations per animal across timepoints. Top right: venn-diagram of animal distribution across time points (n=63 animals).

B) Proportion of morpho-types across each animal.

C) Proportion of phenotypes across timepoints.

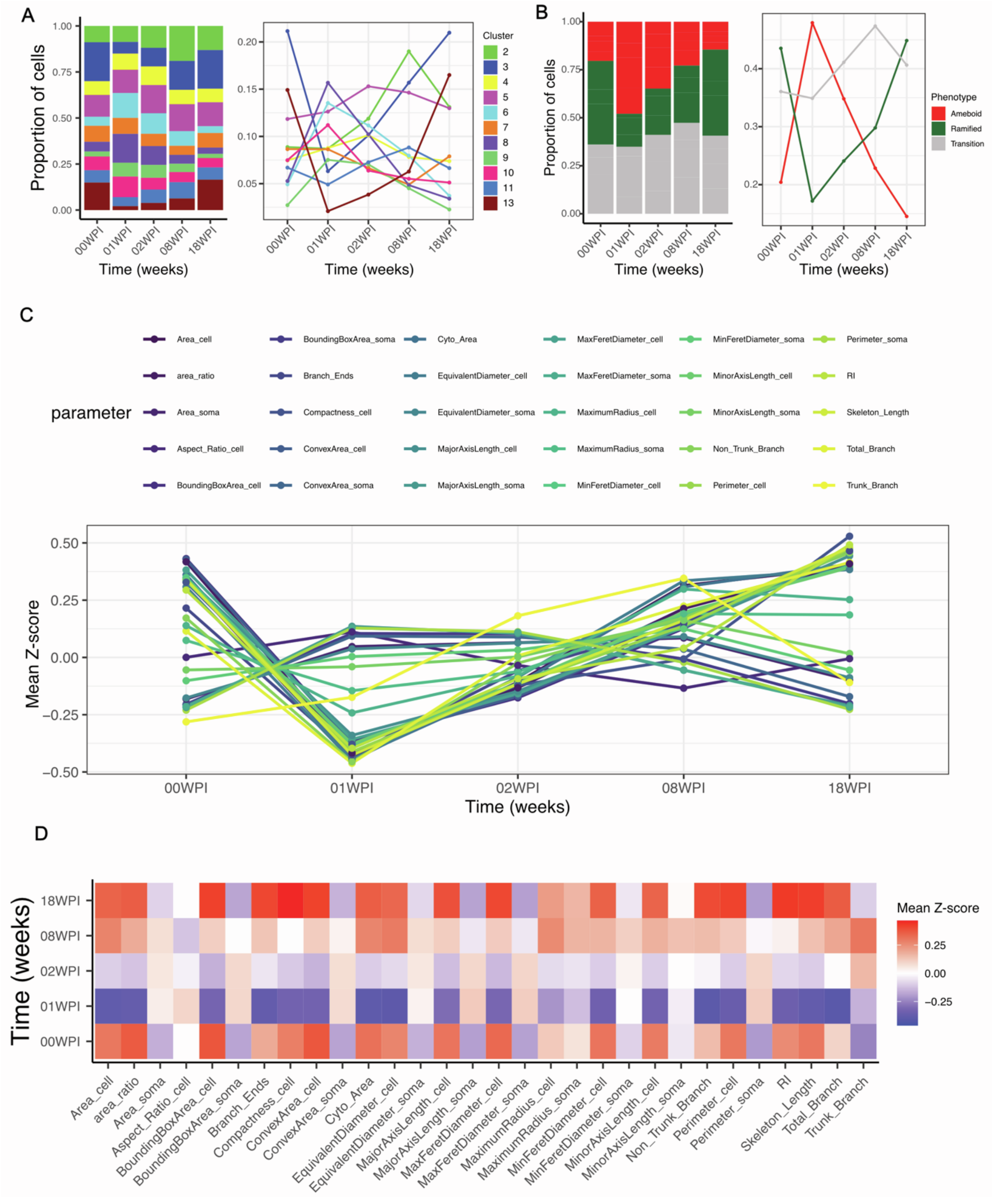

Figure S7 Temporal dynamics of microglial morphotypes following probe implantation

A) (left) Temporal distribution of clusters across implantation time points (00, 01, 02, 08, and 18WPI). (right) Lines represent the trend of cell proportions assigned to each cluster at each time point.

B) (left) Temporal distribution of phenotypes across implantation time points (00, 01, 02, 08, and 18WPI). (right) Lines represent the trend of cell proportions assigned to each phenotype at each time point.

C) Z-score line trend of morphometric parameters across time points showing coordinated temporal shifts in cellular and somatic features.

D) Heatmap illustrating parameter trends across time points.

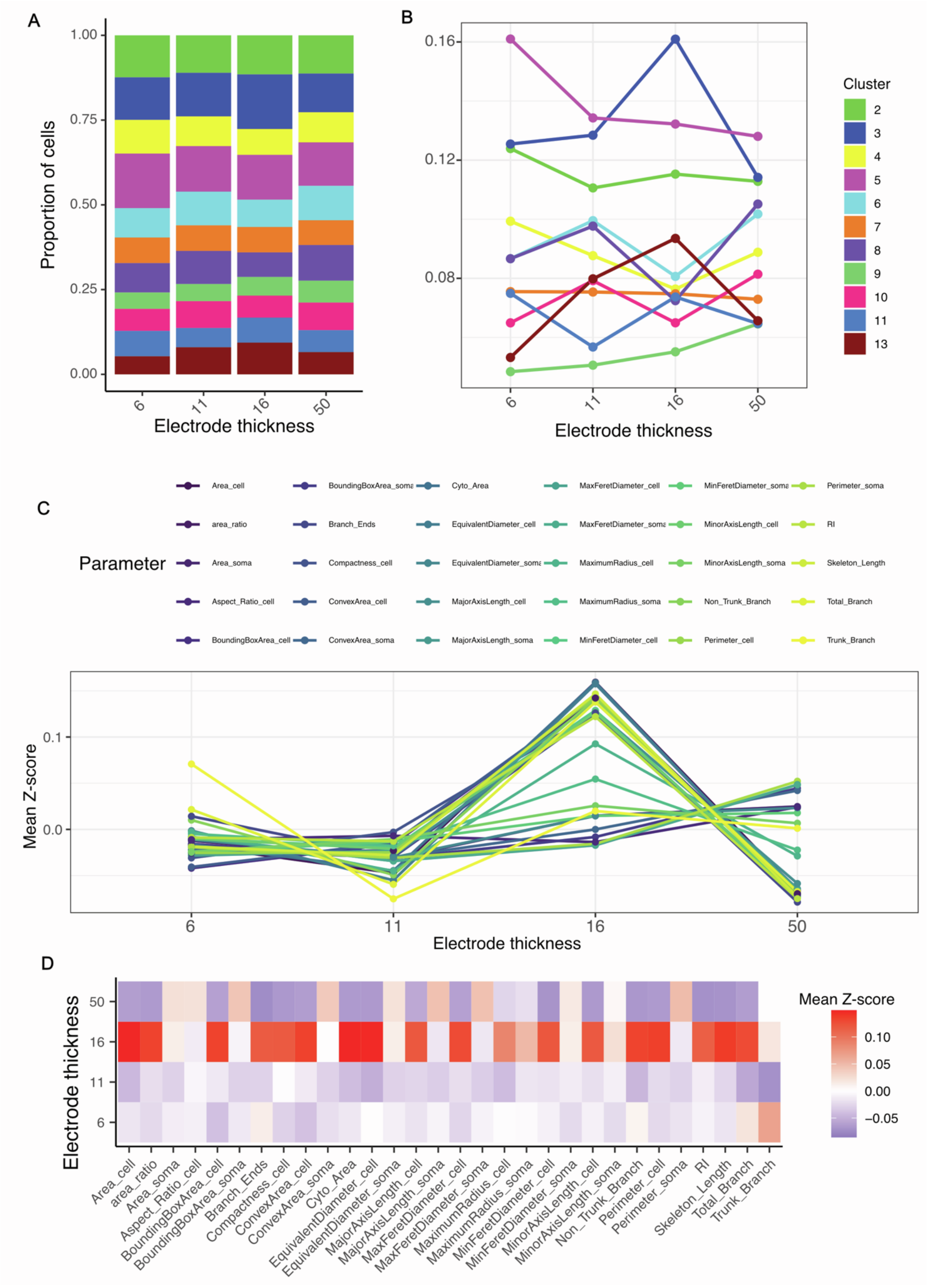

Figure S8 Probe thickness does not substantially alter morphotype dynamics

A-B) Comparison of cluster proportions across different probe thickness(μm).

C-D) Z-score profiles of morphometric parameters across electrode sizes. Comparison of parameter trends across probe thickness conditions, showing largely consistent temporal

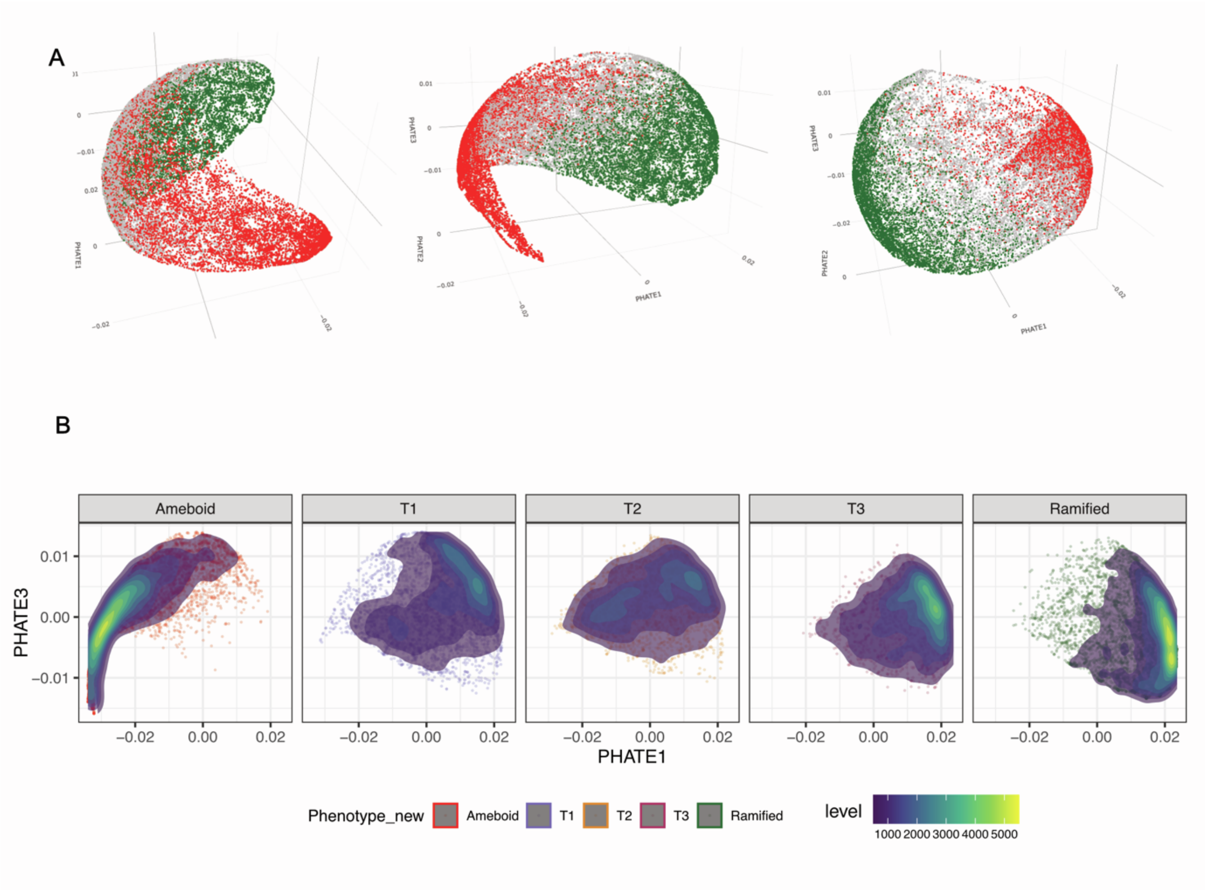

Figure S9 Additional characterization of transitional morphotypes

A) 3D PHATE visualization highlighting the spatial distribution of phenotypes

B) Density plots of PHATE1 vs PHATE3 dimensions showing distinct separation between the phenotypes (ramified, T1, T2, T3, and ameboid).

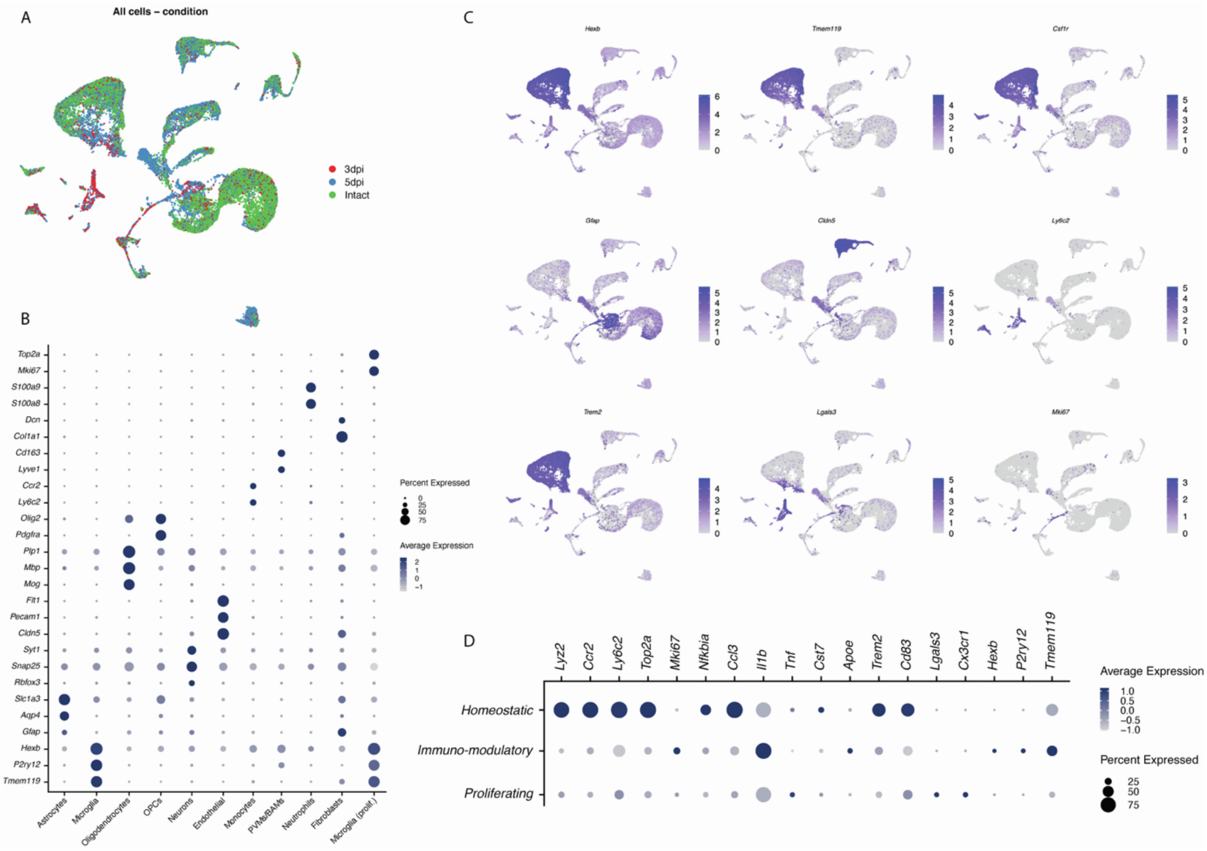

Figure S10 Cell-type annotation and marker validation in the cortical stab wound dataset (GSE226211).

A) UMAP projection of all cells colored by condition (intact, 3 dpi, 5 dpi), showing integration across time points following RPCA-based batch correction.

B) Dot plot of canonical marker gene expression across major cell types used for annotation. Dot size represents the fraction of cells expressing each gene, and color intensity represents average expression. Markers include microglial (*Hexb, Tmem119, P2ry12*), oligodendrocyte (*Mbp, Mog, Plp1*), astrocyte (*Aqp4, Gfap*), endothelial (*Pecam1, Cldn5*), monocyte/macrophage (*Ccr2, Ly6c2, Cd163*), and proliferative (*Top2a, Mki67*) signatures.

C) Feature plots showing expression of representative lineage and state markers across the UMAP, including microglial identity markers (*Hexb, Tmem119, Csf1r*), astrocytic (*Gfap*), endothelial (*Cldn5*), monocyte-associated (*Ly6c2*), proliferative (*Mki67*), and disease-associated microglia (DAM) markers (*Trem2, Lgals3*).

D) Dot plot of marker expression across microglial subtypes (Homeostatic, Immunomodulatory, Proliferating). The Immunomodulatory population shows enrichment of DAM-associated genes (*Lgals3, Trem2, Apoe, Cst7*) and inflammatory mediators, while Homeostatic microglia retain higher expression of canonical identity markers (*P2ry12, Tmem119*). Proliferating microglia are characterized by cell cycle gene expression (*Mki67, Top2a*).

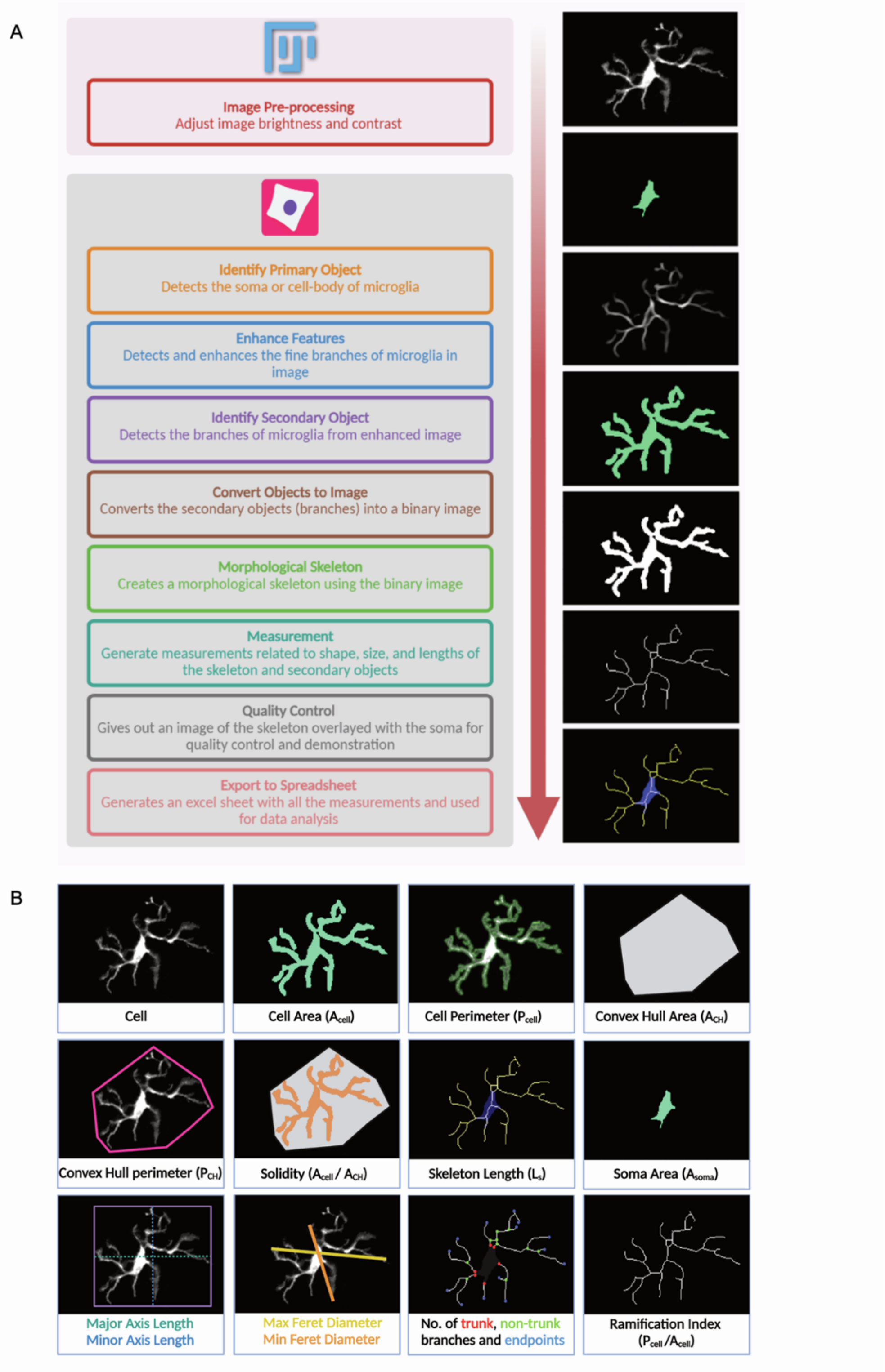

Figure S11 Detailed flow chart of MicroFace

A) Microglia skeleton segmentation using custom made pipeline with module description. On the left side we see all the modules used in the process. The process involved two software tools, FIJI and CellProfiler, with specific modules assigned to each. Pink blocks denote modules executed in FIJI, while grey blocks represent those carried out in CellProfiler. Each module is graphically represented for easy visualization.

B) Illustrations and formulas of the morphological parameters utilized to quantify microglial cells.

SUPPLEMENTARY TABLES

Animal_ID	Total Cells	No. of images
1	3637	4
2	2846	4
3	3247	4
4	1008	1
5	3048	4
6	3654	4
7	3337	4
8	3961	4
9	4037	4
10	3360	4
11	2848	4
12	3476	4
13	3448	4
14	6100	4
15	5501	4
16	4414	3
17	4447	4
18	2698	1
19	1399	4
20	5695	4
21	5404	4
22	7880	4
23	7282	4
24	5593	4
25	5546	4
26	5639	4
27	7730	4
28	6244	4
29	7664	4
30	5796	4
31	6513	4
32	9092	4
33	5928	4
34	7652	4
35	7202	4

36	7973	4
37	7433	4
38	4821	3
39	7298	4
40	5597	4
41	5014	4
42	2130	2
43	2185	2
44	4815	4
45	4540	4
46	5049	4
47	5130	4
48	708	1
49	2233	2
50	2065	3
51	2770	4
52	2981	4
53	770	1
54	4393	4
55	3489	4
56	3086	3
57	2871	3
58	3415	4
59	4121	4
60	1730	2
61	2914	3
62	3467	4
63	3186	4

Table S2 Metadata table showing total number of cells per animal

**Parameter**	**Description**
Number of branches	The number of branches in the skeleton
Number of endpoints	The number of endpoints (i.e. tips) in the skeleton
Total branch length	The sum of the lengths of all branches
Average branch length	The mean length of branches
Branch length standard deviation	The standard deviation of the lengths of branches
Area	The total area of the object
Perimeter	The length of the object's perimeter
Eccentricity	A measure of how elongated the object is
Orientation	The angle between the major axis of the object and the horizontal axis
Solidity	The proportion of the object's pixels that belong to the object (as opposed to holes or background)
Equivalent diameter	The diameter of a circle with the same area as the object
Major axis length	The length of the object's major axis
Minor axis length	The length of the object's minor axis
Form factor	A measure of how closely the object resembles a circle
Convex area	The total area of the smallest convex polygon that encloses the object
Convex perimeter	The length of the perimeter of the smallest convex polygon that encloses the object
Feret diameter	The length of the longest line segment that can be drawn through the object in any direction
Minimum Feret diameter	The length of the shortest line segment that can be drawn through the object in any direction
Feret angle	The angle between the longest line segment through the object and the horizontal axis
Minimum Feret angle	The angle between the shortest line segment through the object and the horizontal axis
Maximum diameter	The length of the longest line segment that can be drawn within the object
Minimum diameter	The length of the shortest line segment that can be drawn within the object
Aspect ratio	The ratio of the length of the major axis to the length of the minor axis
Roundness	A measure of how closely the object resembles a circle, calculated as 4pi(area/perimeter^2)
Compactness	A measure of how compact the object is, calculated as (perimeter^2)/(4pi*area)

Table S3 Name and the definition of the parameters used for morphometric analysis

**Module**	**Description**
Images	Inputs the original images into the pipeline for processing. Supports formats like JPEG, TIFF, etc.
Metadata	Extracts metadata such as image name, capture date, and acquisition details.
NamesAndTypes	Assigns meaningful names and data types (e.g., grayscale, RGB) to images and their channels.
Groups	Groups images based on criteria like size or contrast for efficient processing.
RescaleIntensity	Adjusts image intensity to enhance contrast, brightness, and normalize the intensity range.
IdentifyPrimaryObjects	Identifies primary objects (e.g., microglia soma) using techniques like thresholding or edge detection.
EnhanceOrSuppressFeatures	Enhances or suppresses features like neurites using algorithms like Gaussian or Laplacian of Gaussian filters.
IdentifySecondaryObject	Identifies secondary objects (e.g., microglia branches) using methods like global thresholding.
ConvertObjectToImage	Converts identified objects into binary images for further processing.
MorphologicalSkeleton	Generates skeletons of microglia branches using thinning or skeletonization techniques.
MeasureObjectSkeleton	Measures skeleton features like branch length, count, and number of endpoints.
MeasureObjectShapeSize	Measures soma size and shape features like area, perimeter, and circularity.
calculateilluminationFunction	Calculates the illumination function of the microscope system for correction purposes.
ApplyIlluminationFunction	Applies the calculated illumination function to correct image illumination variations.
ExportToSpreadsheet	Saves measurements of identified objects/features into a spreadsheet for analysis.
SaveImages	Saves processed images or overlays in formats like TIFF, PNG, or JPEG.
OverlayObjects	Overlays identified objects/features on original or other image channels for visual representation.

Table S4 Name and the description of the CellProfiler modules used for MicroFace pipeline.
